# CD56^dim^ CD16^−^ Natural Killer Cell Profiling in Melanoma Patients Receiving a Cancer Vaccine and Interferon-α

**DOI:** 10.3389/fimmu.2019.00014

**Published:** 2019-01-29

**Authors:** Lazar Vujanovic, Christopher Chuckran, Yan Lin, Fei Ding, Cindy A. Sander, Patricia M. Santos, Joel Lohr, Afshin Mashadi-Hossein, Sarah Warren, Andy White, Alan Huang, John M. Kirkwood, Lisa H. Butterfield

**Affiliations:** ^1^University of Pittsburgh Hillman Cancer Center, Pittsburgh, PA, United States; ^2^Department of Medicine, University of Pittsburgh School of Medicine, Pittsburgh, PA, United States; ^3^Department of Immunology, University of Pittsburgh School of Medicine, Pittsburgh, PA, United States; ^4^Department of Biostatistics, University of Pittsburgh School of Medicine, Pittsburgh, PA, United States; ^5^NanoString Technologies, Seattle, WA, United States; ^6^Department of Surgery, University of Pittsburgh School of Medicine, Pittsburgh, PA, United States

**Keywords:** melanoma, CD56^dim^ CD16^−^, natural killer cell subsets, dendritic cells, recombinant adenoviral vector, interferon-α

## Abstract

Natural killer (NK) cells are innate cytotoxic and immunoregulatory lymphocytes that have a central role in anti-tumor immunity and play a critical role in mediating cellular immunity in advanced cancer immunotherapies, such as dendritic cell (DC) vaccines. Our group recently tested a novel recombinant adenovirus-transduced autologous DC-based vaccine that simultaneously induces T cell responses against three melanoma-associated antigens for advanced melanoma patients. Here, we examine the impact of this vaccine as well as the subsequent systemic delivery of high-dose interferon-α2b (HDI) on the circulatory NK cell profile in melanoma patients. At baseline, patient NK cells, particularly those isolated from high-risk patients with no measurable disease, showed altered distribution of CD56^dim^ CD16^+^ and CD56^dim^ CD16^−^ NK cell subsets, as well as elevated serum levels of immune suppressive MICA, TN5E/CD73 and tactile/CD96, and perforin. Surprisingly, patient NK cells displayed a higher level of activation than those from healthy donors as measured by elevated CD69, NKp44 and CCR7 levels, and enhanced K562 killing. Elevated cytolytic ability strongly correlated with increased representation of CD56^dim^ CD16^+^ NK cells and amplified CD69 expression on CD56^dim^ CD16^+^ NK cells. While intradermal DC immunizations did not significantly impact circulatory NK cell activation and distribution profiles, subsequent HDI injections enhanced CD56^bright^ CD16^−^ NK cell numbers when compared to patients that did not receive HDI. Phenotypic analysis of tumor-infiltrating NK cells showed that CD56^dim^ CD16^−^ NK cells are the dominant subset in melanoma tumors. NanoString transcriptomic analysis of melanomas resected at baseline indicated that there was a trend of increased CD56^dim^ NK cell gene signature expression in patients with better clinical response. These data indicate that melanoma patient blood NK cells display elevated activation levels, that intra-dermal DC immunizations did not effectively promote systemic NK cell responses, that systemic HDI administration can modulate NK cell subset distributions and suggest that CD56^dim^ CD16^−^ NK cells are a unique non-cytolytic subset in melanoma patients that may associate with better patient outcome.

## Introduction

Malignant melanoma is the most lethal cutaneous cancer that causes the majority of skin cancer-related deaths (80%). It is a neoplasm of melanocytes that commonly initiates in the skin and eye. Early-stage, localized lesions are normally treatable with surgical excision (5-year survival rate of 90–100% for stage 0/I). Unfortunately, due to its invasive properties, the median survival for individuals with metastatic disease is only 6–10 months (1). Recent advances in molecular biology and immunology have led to the identification of a number of promising therapeutic targets, with therapies that engage these targets having become a focus of drug development for melanoma. Among these, therapies targeting mitogen-activated protein kinase (MAPK) signaling pathway in lesions that harbor mutated BRAF kinase and negative regulatory checkpoint molecules of the immune system [cytotoxic T-lymphocyte antigen-4 (CTLA-4) and programmed death receptor-1 (PD-1)] became standards of care for metastatic melanoma (1–3). Clinical benefit of these therapies has been limited to a subset of patients, stressing the need for novel therapeutic modalities to treat melanoma. Cancer vaccines, such as dendritic cell (DC)-based vaccines could be one such strategy.

DC are professional antigen presenting cells that can effectively activate naïve CD4+ and CD8+ T cell responses as well as Natural Killer (NK) cells, and play a key role in host immune responses against infections and cancers (4). DC-based vaccines have been shown to be safe, immunogenic and capable of promoting long-term tumor-specific immunity, survival and durable objective clinical responses in a minority of patients [4.2–7.1%; (5–8)]. Our lab has developed a novel autologous DC-based vaccine capable of inducing adaptive and innate anti-tumor immunity *in vitro*. We engineered lipopolysaccharide (LPS) and interferon-γ (IFNγ)-matured, type-1-skewing DCs to express three melanoma tumor antigens (tyrosinase, MART-1 and MAGE-A6) through replication-defective adenovirus (AdV) transduction (9–11). The vaccines were administered to patients at three time-points, and patients were subsequently randomized to observation or to receive boost of 1 month of high-dose systemic interferon-α2b (IFNα; HDI). IFNα is a type I interferon which is a potent modulator of immune responses. It can promote strong type-1 anti-tumor T cell responses and is a major regulator of NK cell-mediated cytotoxicity (12–15). Because IFNα has been shown to have a significant benefit on the overall survival and prolonged relapse-free survival, it is commonly used as an adjuvant immunotherapy for melanoma (16). The clinical trial results revealed a clinical response rate of 6% and increased vaccine antigen recognition by T cells in the majority of vaccinated patients. HDI adjuvant therapy did not enhance T cell or clinical responses (Butterfield et al., under review).

Because DC have been shown to be potent inducers of NK cell activation by elevated expression of activation markers as well as enhanced IFNγ secretion, lytic activity and proliferation, we investigated whether the DC ± IFNα strategy also enhanced NK cell activity in melanoma patients (17, 18). NK cells are an important class of innate cytotoxic lymphocytes which mediate killing of virally-infected or transformed cells. Their function is regulated by an elegant interplay of numerous activating and inhibitory surface receptors that are either specific for self-peptides expressed on healthy cells or for molecules that are upregulated during stress (19, 20). Consequently, missing self-antigens, marked overexpression of normal self-peptides, or the expression of altered self-peptides can stimulate NK cell cytotoxicity (21). The integration of multiple simultaneous receptor-ligand interactions and cytokine signals determines NK cell activation as well as functional response (19, 20). Depending on context, NK cells have the ability to act as either immunoregulators through cytokine production or direct effector cells (20).

In human cancer, NK cells play a significant role in both killing of transformed cells as well as immune regulation through the production of cytokines (20, 22, 23). Decreased patient NK cell function has been reported in hepatocellular carcinoma, head and neck cancer, breast cancer, and melanoma (24–27). A seminal 11-year observational study suggested that increased peripheral blood NK cell lytic activity correlated with decreased cancer incidence (28). In the melanoma setting, it is generally believed that NK cell dysfunction correlates with reduced tumor immunosurveillance and increased cancer susceptibility (22, 23, 29–31). However, a recent study has indicated that tumor-draining lymph nodes generate and/or recruit highly cytotoxic CD56^dim^ NK cells, indicating that NK cell functions are not as suppressed in melanoma patients as previously believed (32).

We have shown that AdV-transduced DC (AdV.DC) recruit CD56^dim^ CD16^+^ and CD56^bright^ CD16^−^ NK cell subsets via CXCL8/IL-8 and CXCL10/IP-10, respectively, and activate NK cell Th1 functions through cell contact-mediated interactions of membrane-bound tumor necrosis factor (TNF) and IL-15 (18, 33). We have also shown that combination of IFNα with AdV.DC further enhances NK cell activation and cytotoxicity *in vitro* (11). Based on these data, we examined the impact of intradermal AdV.DC ± systemic HDI administration on peripheral blood NK cell profiles in melanoma patients. We characterized differences in immunosuppressive serum factors, NK cell cytotoxicity, phenotype, and subpopulation distribution between patients with and without measurable disease and healthy donor controls in blood, and profiled subpopulation distributions of tumor-infiltrating NK cells (TINKs).

## Materials and Methods

### Antibodies

NK cell phenotype of melanoma patients enrolled in the trial was examined using fluorochrome-conjugated antibodies against the following cell-surface markers: CD56-FITC, CD3-PC7, CD16-APC, CD69-BV421, NKp30-BV711, CXCR3-BV421, CCR3-BV510 (BD Biosciences; San Diego, CA), NKp44-PerCP eFluor 710 (eBioscience; San Diego, CA), CXCR1-PE (R&D Systems; Minneapolis, MN), CCR7-BV711 (BioLegend; San Diego, CA), and matching IgG isotype controls from the same vendors. The immune checkpoint and NK cell activation receptor panel included the following markers: Zombie NIR Fixable Viability Dye (BioLegend; San Diego, CA), CD3-PE-Vio770 (Miltenyi Biotec; San Diego, CA), ANK-1-PE (Santa Cruz Biotechnology; Dallas, TX), TIGIT-PerCP eFluor 710 (eBioscience), CD45-BUV395, CD56-BV510, CD16-BUV737, NKG2D-APC, NKp46-BV711, CD69-BV421, and PD-1-BV650 (BD Biosciences).

### Patients and Their Treatments

This was a Phase I, single site study to evaluate the immunological effects of autologous DC transduced with the MART-1, tyrosinase and MAGE-A6 genes in 35 subjects with recurrent, unresectable stage III or IV melanoma (M1a, b, or c), or resected stage IIIB-C or IV melanoma (Supplemental Table 1). 5 × 10^6^-10^7^ AdV.DC were given intradermally every 2 weeks for a total of 3 vaccines. After the AdV.DC immunizations, subjects were randomized to either receive a boost of HDI or no boost. Subjects randomized to receive the IFNα boost received Interferon-α2b (Intron A, Schering-Plow), 20 MU/m2/d (rounded to the nearest 1 million units) administered intravenously for 5 consecutive days (Monday through Friday) every week for 4 weeks. Administration began approximately 30 days (±7 days) after the 3rd vaccine (Butterfield et al., under review).

### Patient Sample Acquisition and Storage

With informed consent, peripheral blood and tumor biopsies were obtained from healthy donor (HD) and melanoma patients (HCC #04-001, #09-021 and #96-099). Patient characteristics are described in Supplemental Tables 1, 2. Peripheral blood mononuclear cells (PBMCs) were separated from HD blood using Ficoll Hypaque gradient centrifugation (Corning, Manassas, VA) as previously described (34) and cryopreserved as aforementioned. Monocytes and lymphocytes isolated by elutriation from the baseline, day 43 and day 89/101 leukaphereses were cryopreserved in 50% RPMI, 40% HuAB serum (Gibco; Fisher Scientific; Waltham, MA) and 10% DMSO (Sigma). A red top tube (no anticoagulant) was also drawn at each time point for serum to test for cytokine/chemokine/growth factor/immunosuppressive factor levels. Patient samples were acquired in parallel with a HD control sample. Serum was clotted at room temperature, aliquoted, and frozen at −80°C. Serum was kept in a monitored freezer and tested after a single thaw. Bulk melanoma single cell suspensions were collected and cryopreserved as previously reported (35). All patient specimens were processed by competency-trained technologists under standard operating protocols in the Immunologic Monitoring Laboratory.

### NK Cell Isolation and Culture

Cryopreserved patient lymphocyte and HD PBMC samples were thawed using RPMI + 10% FBS media supplemented with 0.5% DNAse (Sigma) and immediately prepared for analysis and testing. Thawed cell viabilities were between 65 and 92% as measured by trypan blue exclusion (Gibcol Fisher Scientific), with the mean viability of 81%. One portion of cells was used for multi-color flow cytometric analysis of NK cells. NK cells were purified from the remaining cells by negative magnetic cell sorting selection using the Human NK cell Isolation Kit (Miltenyi Biotec). NK cells were cultured in AIM-V medium supplemented with 5% human AB serum. NK cells were resuspended in AIM-V media (Gibco; Fisher Scientific) at a concentration of 5.0 × 10^6^ cells/mL.

### Cell Line

K562 erythroleukemia cell line was obtained from ATCC in 2000, and was authenticated by flow cytometry. K562 was cultured in RPMI 1640 medium, supplemented with 10% fetal bovine serum, 1% penicillin-streptomycin, and 1% L-glutamine (Life Technologies), at 37°C, in a humidified 5% CO_2_ atmosphere. It was passaged bi-weekly, and was used in the described experiments after every other passage. The cell line was negative for Mycoplasma contamination as shown by GEN-PROBE Mycoplasma Tissue Culture Non-Isotopic Rapid Detection System (Gen-Probe, Inc.; San Diego, CA).

### NK-Target Cell Visualization Assay (NK-TVA)

Target cells (K562) were cytosolically labeled with TVA dye (Cell Technologies Ltd.; Shaker Heights, OH) and co-cultured with isolated patient NK cells for 3 h at 37°C and 5% CO_2_. NK:K562 ratios used were 100:1, 50:1, 25:1. 12.5:1, 6.25:1, 3:1, 1.5:1, and 0:1. Killing was measured by counting the remaining target cells after co-culture using an ImmunoSpot S6 ULTIMATE analyzer (Cell Technologies Ltd.). The percentage of cytotoxic activity and lytic units (LU)_20_/10^7^ effector cells were calculated as previously reported (36).

### Absolute Cell Counts

One heparinized tube of whole blood was drawn at each time point from each patient for fresh whole blood flow cytometry to obtain absolute counts and percentages of PBMC subsets (Beckman TQ-prep, Beckman Coulter, Brea, CA). Cells were stained for CD3, CD16, and CD56. Flow cytometry was performed on a Coulter FC500 and analyzed by CXP software (Beckman Coulter) (Butterfield et al., under review).

### Flow Cytometry

One-step staining of cell-surface antigens was performed using fluorochrome-conjugated primary antibodies as previously described (10, 18, 33). For the analysis of blood NK cells two antibody panels were constructed around CD56-FITC, CD16-APC, and CD3-PE-Cy7 (BD Bioscience, San Jose, CA) antibodies. NK cell activation receptors were evaluated with CD69-BV421, NKp30-BV711 (BD Bioscience), and NKp44-PerCP eFluor 710 (eBioscience, San Diego, CA) antibodies. Chemokine expression levels were tested using CXCR1-PE (R&D Systems, Minneapolis, MN), CXCR3-BV421, and CCR7-BV510 (BD Bioscience) antibodies. For the TINK analysis, Zombie NIR (BioLegend), CD45 BUV395, CD56 BV510, CD16 BUV737 (BD Bioscience), and CD3 PE-Vio770 (Miltenyi Biotec) antibodies were used. Suitable IgG controls were acquired from the same vendors. FACS analyses were performed using the BD LSRFortessa™ cell analyzer, and analyzed using FlowJo v10 (FlowJo, LLC; Ashland, OR) software.

### Serum Testing

Patient and HD sera were assessed for multiple cytokines, chemokines, and immunosuppressive factors using the Cytokine/Chemokine/Growth Factor 45-Plex Human Panel 1 and Immuno-Oncology Checkpoint 14-Plex Human Panel 2 Luminex kits (ThermoFisher, Pittsburgh, PA).

### Multiplex Gene Expression Analysis

Total RNA isolated from patient tumor biopsies (QIAGEN; Valencia, CA) was profiled on the nCounter® MAX platform with the PanCancer Immune Profiling Panel (NanoString Technologies, Inc.; Seattle, WA). Single molecule imaging was performed on the nCounter SPRINT Profiler (NanoString Technologies) and gene expression data were analyzed using the R statistical software. Gene expression data from nCounter platform were log2 transformed and normalized using stable expression of housekeeping genes. Genes and the weights involved in calculations of metagenes were developed a priori by NanoString. Following calculation of the metagene values for each sample, scores were used to evaluate association of each score to patient outcome. The outcome here was defined either as a binary value based on “better” [partial responder (PR), stable disease (SD) and patients with resected tumors and no evidence of disease (NED)] and “worse” (progressive disease) prognosis. Each outcome was regressed against the scores calculated. In the case of the binary outcome, a univariate linear model regressing each score on the binary outcome generated expected log2 ratios of scores for the patients with better prognosis relative to those with worse prognosis. The data discussed in this publication have been deposited in NCBI's Gene Expression Omnibus (37) and are accessible through GEO Series accession number GSE124574 (https://www.ncbi.nlm.nih.gov/geo/query/acc.cgi?acc=GSE124574).

### Statistical Analysis

Two-tailed non-parametric tests were used for all analyses. To explore the relationship between two tests groups [e.g., HD vs. melanoma patients; Day 43 vs. Baseline differences to determine the impact of DC immunizations on the profile of NK cells; and IFNα vs. observation groups], the Wilcoxon rank-sum test was performed. When comparing three groups [e.g., HD vs. patients with NED vs. patients with measurable disease (MD)], Kruskal-Wallis test was used to get the overall *p*-value, followed by the Wilcoxon rank-sum to determine pairwise *p*-values. For correlation plots, statistical significance was estimated by linear correlation. To explore the relationship between NK cell killing ability, NK cell subsets distributions and expression of activation markers on these subsets, Spearman's correlation was used. Graphs were generated using GraphPad Prism v6 (GraphPad Software, Inc., La Jolla, CA). *P* values < 0.05 were considered to be statistically significant.

## Results

### Late-Stage Melanoma Patient Sera Contain Elevated Levels of Multiple Factors That Suppress NK Cell Function

**Figure 1:**
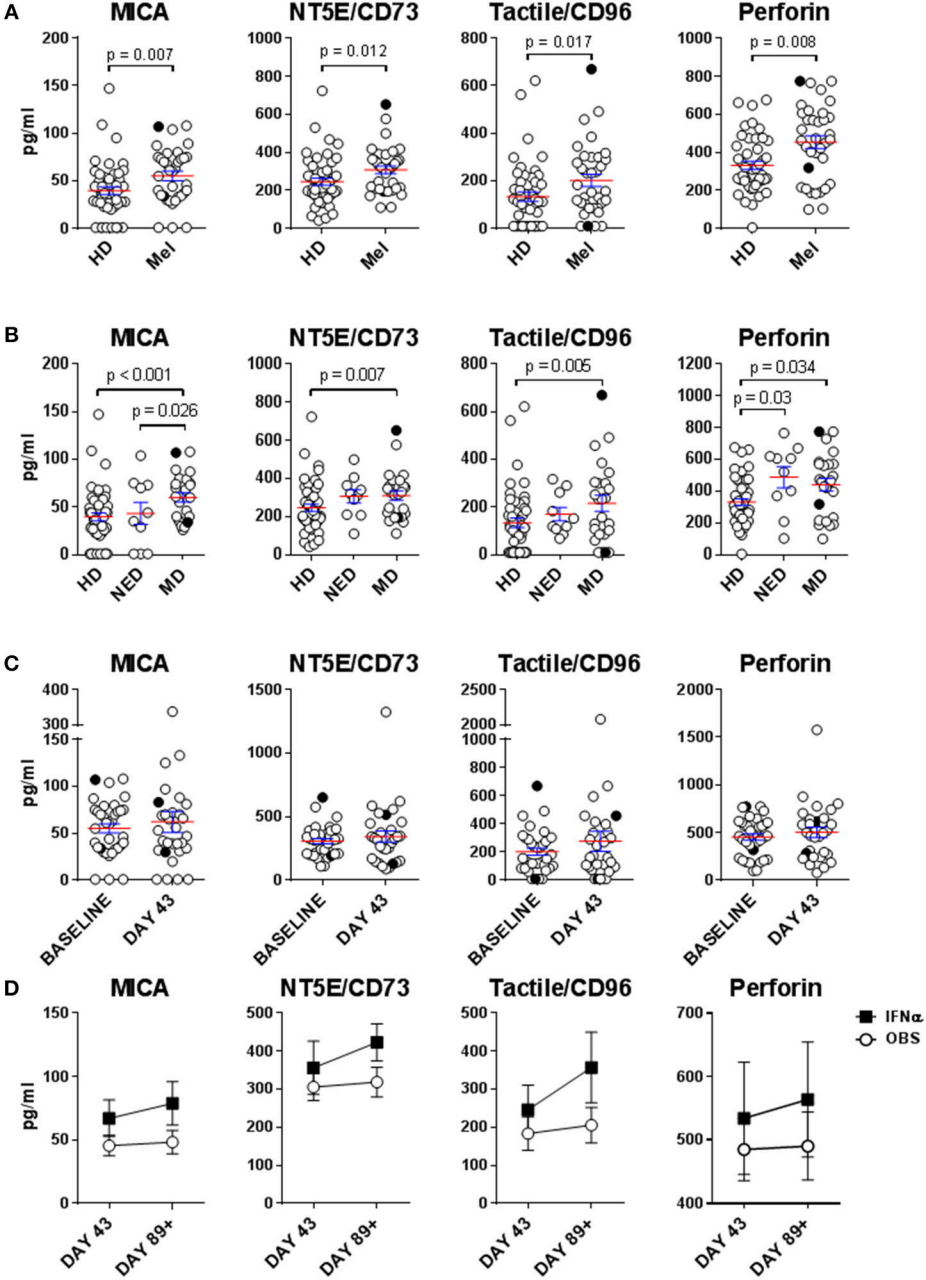
Evaluation of immunosuppressive factors in donor sera. Presence of soluble immunosuppressive factors in sera isolated from healthy donors (HD) and melanoma patients (Mel) was measured by Luminex. **(A,B)** Mel sera isolated prior to treatments (Baseline) were evaluated **(B)** Patients with resected (NED) and unresectable (measurable disease; MD) tumors were compared for their serum concentrations of immunosuppressive factors to HDs. **(C)** Mel sera isolated prior to all the treatments (Baseline) and after DC immunizations (Day 43) are compared. **(D)** Mel sera isolated post DC immunizations are tested in patients in the observation arm of the study and those that received IFNα therapy (IFNα). Intersecting lines and whiskers represent mean and standard error of mean values, respectively. Black circles **(A–C)** represent patients that were PR.

NK cell dysfunction has generally been associated with late-stage melanoma. Multiple mechanisms of NK cell suppression in the cancer setting have been described, including those mediated by tumor- or immune cell-derived soluble factors (30, 38). HD and melanoma patient sera were tested for immunosuppressive factors MICA, NT5E/CD73 and tactile/CD96, as well as perforin, which is released by cytotoxic NK and T cells and is a surrogate biomarker for *in vivo* cytolytic activity of these effectors (39). MICA, NT5E/CD73 and tactile/CD96 showed statistically significant increase in melanoma patients vs. HDs (Figure 1A). These analytes were tumor burden-dependent as NED had similar serum levels of these factors to HDs (Figure 1B). Surprisingly, elevated perforin presence was concurrently observed in melanoma patient sera irrespective of their disease burden possibly indicating aberrant lymphocytic cytolytic activity (Figures 1A,B) indicating enhanced cytolytic activity of NK and T cell effectors (40, 41). None of these analytes were affected by DC immunizations or HDI (Figures 1C,D).

### Circulatory NK Cells Isolated From Late-Stage Melanoma Patients Display an Elevated Level of Activation

Human NK cells can be segregated into at least three major subsets based on their CD56 (NCAM) and CD16 (FcγRIII) expression levels: CD56^bright^ CD16^−^, CD56^dim^ CD16^+^, and CD56^dim^ CD16^−^ NK cells. These subsets have unique phenotypic and functional profiles. NKp30 and CD69 expression levels are upregulated on all three subsets upon activation, but are also present at low levels on resting NK cells. In contrast, NKp44 is exclusively expressed on activated NK cells (33, 42). These three subsets also have specific chemokine receptor profiles which dictate their specific chemokine preferences. We have previously shown that CCR7 is expressed on all three NK cell subsets, CXCR3 is the dominant receptor expressed on CD56^bright^ CD16^−^ NK cells, and CXCR1 is the central receptor presented on CD56^dim^ CD16^+^ NK cells. CD56^dim^ CD16^−^ NK cells express a variety of chemokine receptors, including CXCR1 and CXCR3 (33). NK cell chemokine receptors have been reported to be upregulated following activation (43). We performed a phenotypic analysis of fresh whole-blood NK cells, as well as a more detailed multicolor analysis of cryopreserved lymphocytes (Supplemental Figure 1).

**Figure 2:**
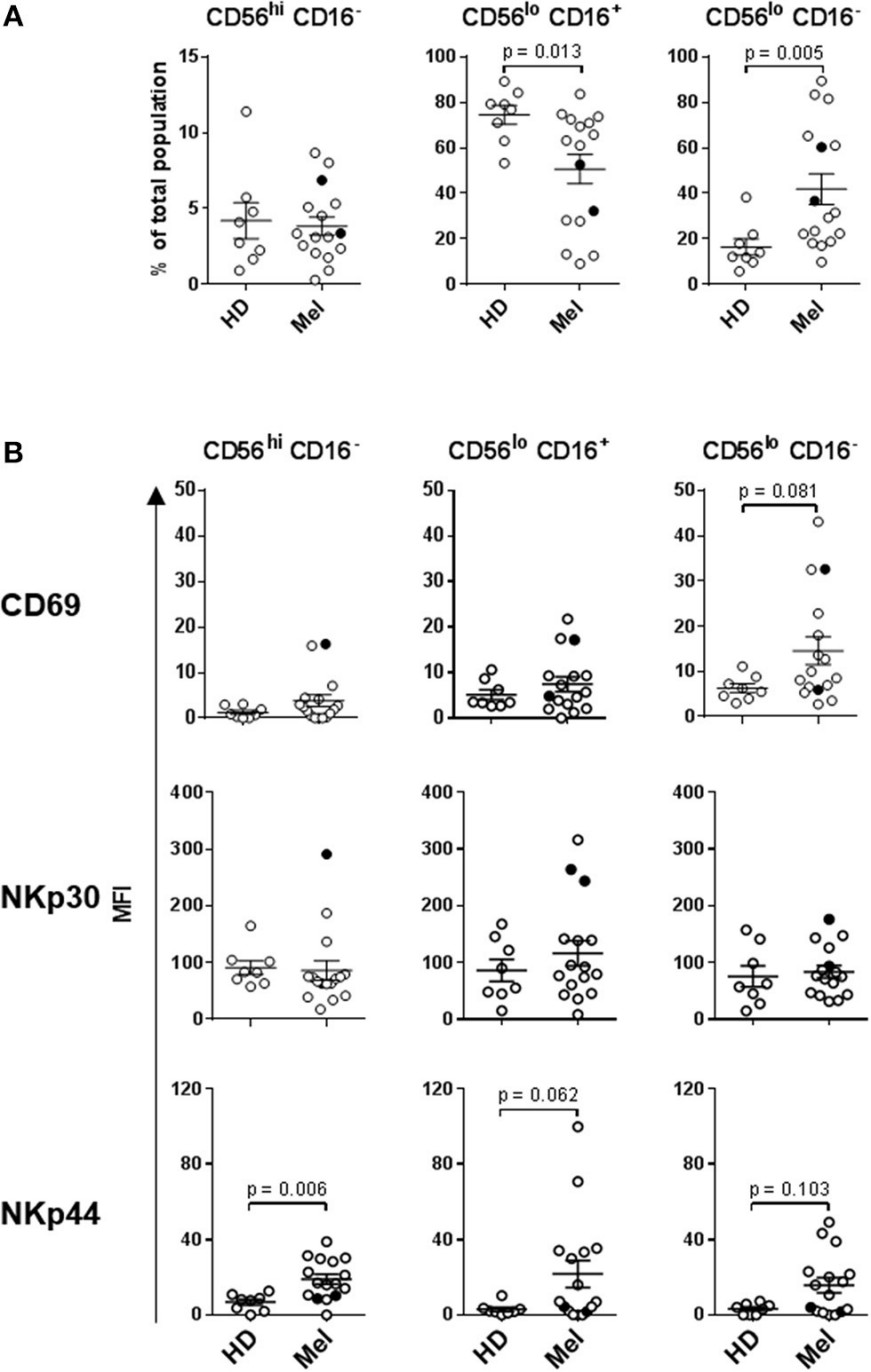
NK cell subset distributions and expression of activation markers. **(A)** Distribution of the three major NK cell subsets based CD56 and CD16 expression levels are shown for HD and Mel (Mel). **(B)** CD69, NKp30, and NKp44 expression levels were measured on the three NK cell subsets isolated from HDs and Mel prior to treatments (Baseline). Intersecting lines and whiskers represent mean and standard error of mean values, respectively. Black circles represent patients that were PR.

**Figure 3:**
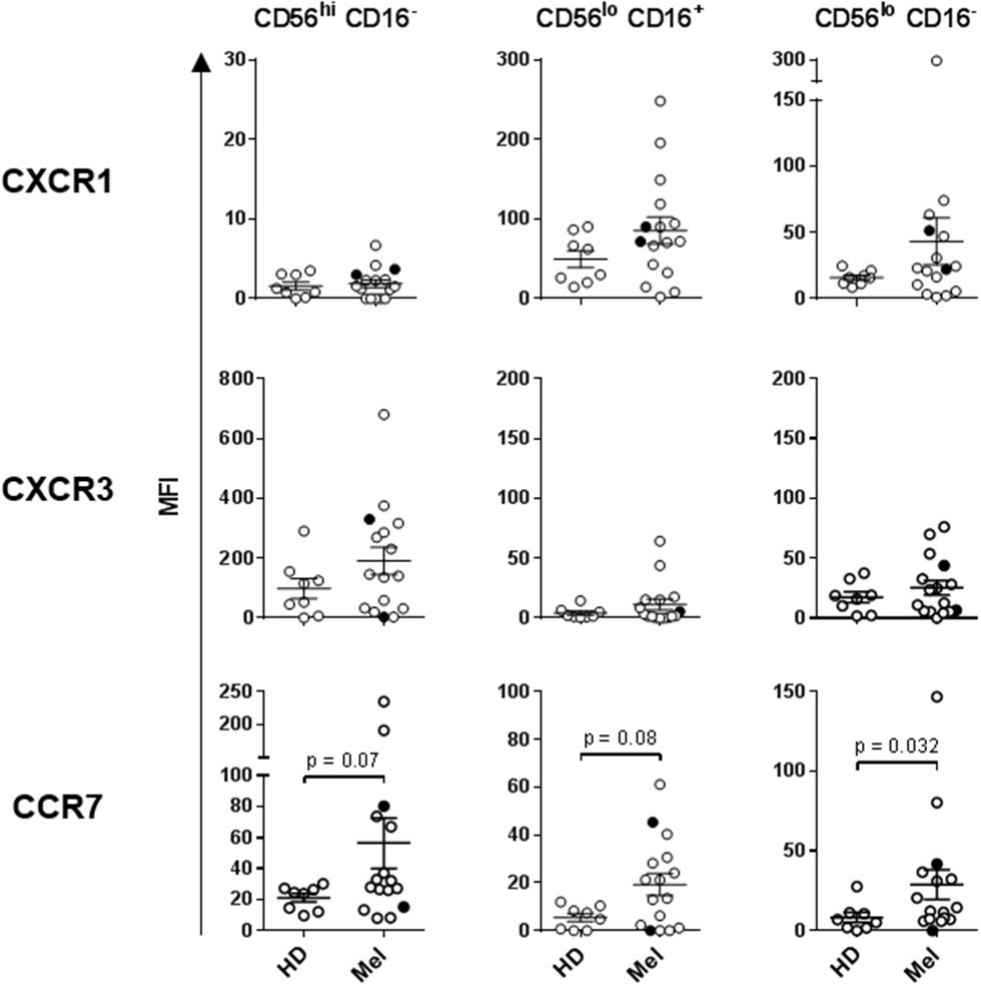
Expression of chemokine receptors on the three NK cell subsets. CXCR1, CXCR3, and CCR7 expression levels were measured on the three NK cell subsets isolated from HDs and melanoma patients prior to treatments (Baseline). Intersecting lines and whiskers represent mean and standard error of mean values, respectively. Black circles represent patients that were PR.

Statistically significant differences in absolute counts of HD and melanoma patient NK cell subsets were not observed, nor were counts significantly affected by DC and HDI treatments (Supplemental Figure 2). A more robust phenotypic evaluation of cryopreserved systemic lymphocytes showed a distribution imbalance of CD56^dim^ CD16^+^ and CD56^dim^ CD16^−^ NK cells in a cohort of melanoma patients, particularly NED patients (Figure 2A, Supplemental Figure 3A). Patient NK cells also displayed elevated level of activation in comparison to HDs. Average expression levels of CD69, NKp44, CXCR1, CXCR3, and CCR7 were elevated on multiple patient NK cell subsets. Of those, CD69, NKp44, and CCR7 showed trends toward increased expression on one or more NK cell subsets (Figures 2B, 3). NKp30 was the only surface protein that showed little to no modulation on any of the melanoma patient NK cell subsets (Figure 2B, Supplemental Figure 3B). Expression of NKp44 was particularly affected by tumor burden as NED patients showed elevated expression on all of the NK cell subsets (Supplemental Figures 3B, 4).

### HDI, but Not DC Vaccines Impacted Melanoma Patient NK Cell Subsets

**Figure 4:**
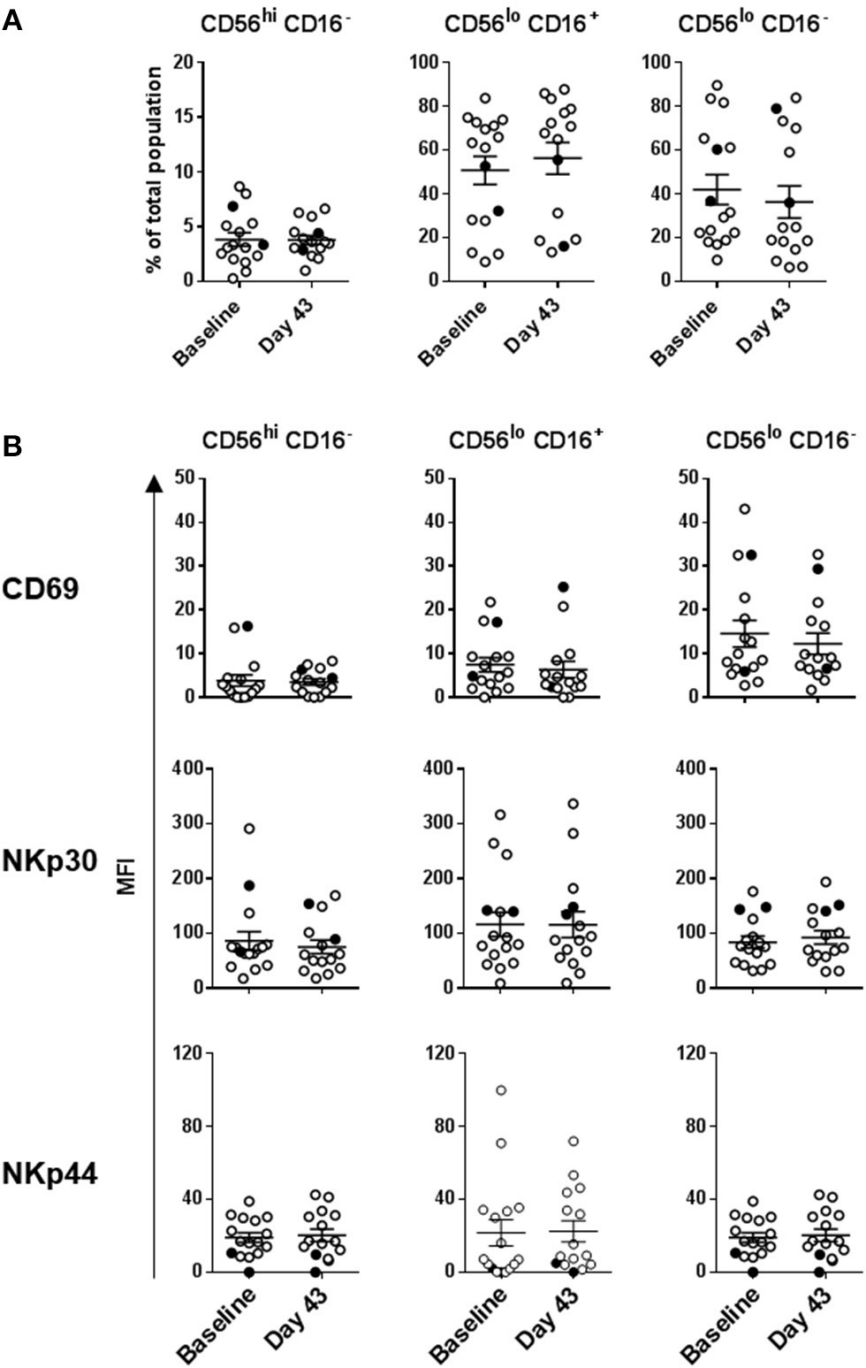
DC immunization impact on NK cell subset distributions and expression of activation markers. **(A)** Distribution of the three major NK cell subsets based CD56 and CD16 expression levels; and **(B)** CD69, NKp30, and NKp44 expression levels measured on the three NK cell subsets are shown for melanoma patients prior to the treatments (Baseline) and after three rounds of DC immunizations (Day 43). Intersecting lines and whiskers represent mean and standard error of mean values, respectively. Black circles represent patients that were PR.

**Figure 5:**
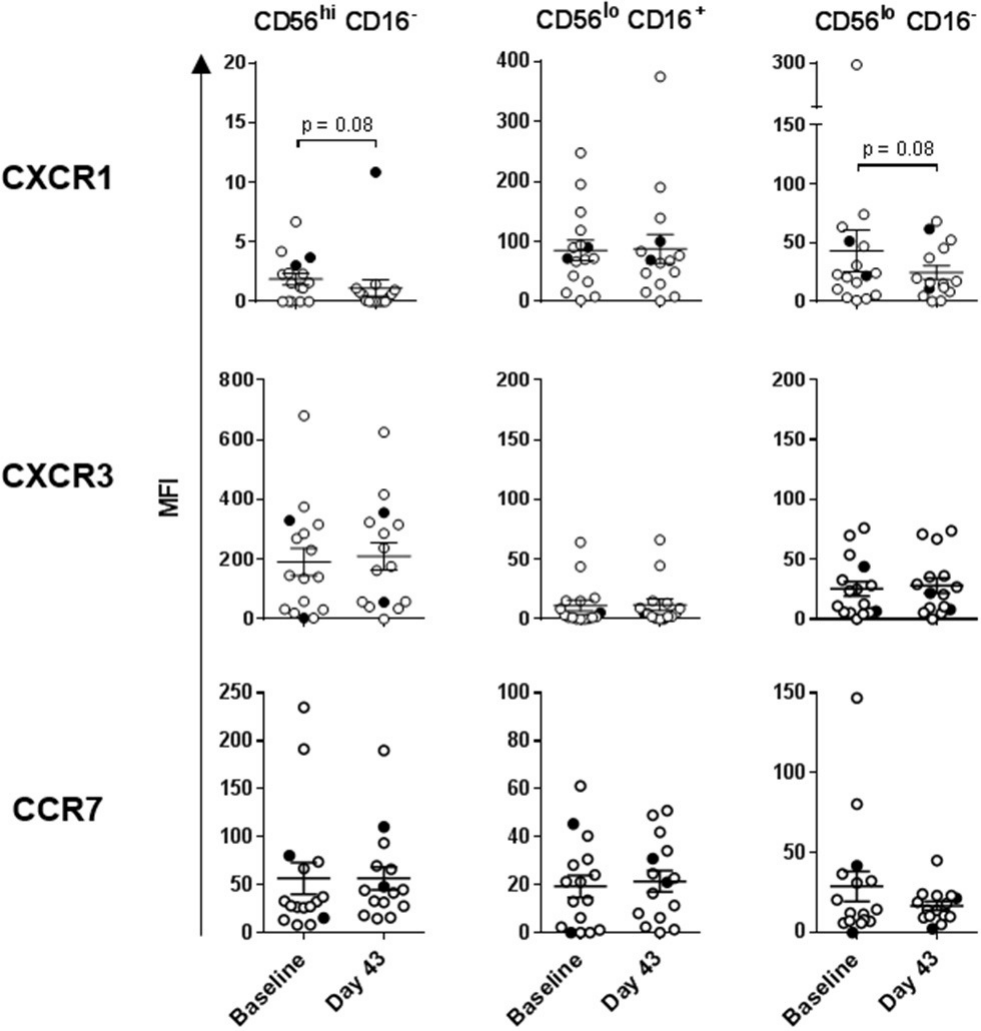
DC impact on expression of chemokine receptors on the three NK cell subsets. CXCR1, CXCR3, and CCR7 expression levels measured on the three NK cell subsets are shown for melanoma patients prior to the treatments (Baseline) and after three rounds of DC immunizations (Day 43). Intersecting lines and whiskers represent mean and standard error of mean values, respectively. Black circles represent patients that were PR.

**Figure 6:**
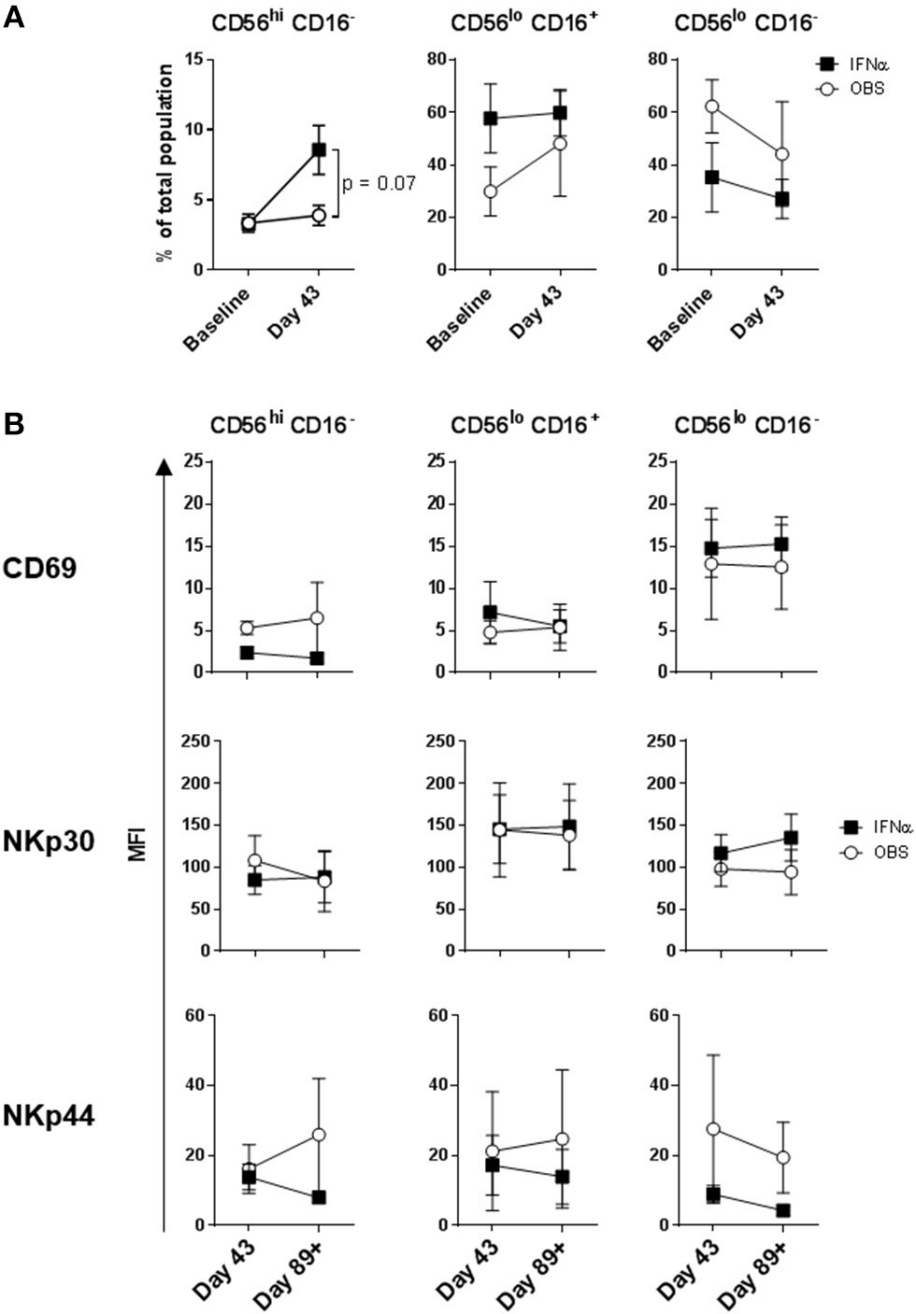
Systemic IFNα therapy impact on NK cell subset distributions and expression of activation markers. **(A)** Distribution of the three major NK cell subsets based CD56 and CD16 expression levels; and **(B)** CD69, NKp30, and NKp44 expression levels measured on the three NK cell subsets are shown for melanoma patients following three rounds of DC immunizations (Day 43) and after HDI (Day 89+). Intersecting lines and whiskers represent mean and standard error of mean values, respectively.

**Figure 7:**
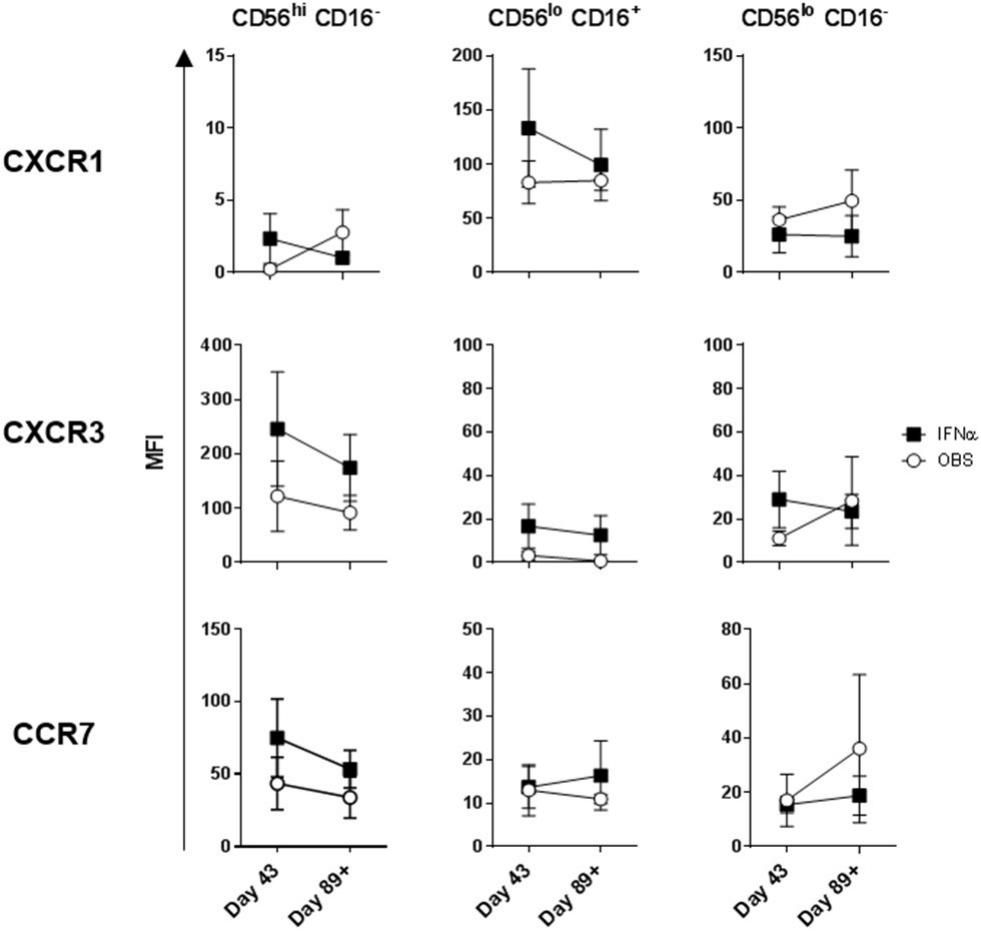
Systemic IFNα therapy impact on expression of chemokine receptors on the three NK cell subsets. CXCR1, CXCR3, and CCR7 expression levels measured on the three NK cell subsets are shown for melanoma patients following three rounds of DC immunizations (Day 43) and after HDI (Day 89+). Intersecting lines and whiskers represent mean and standard error of mean values, respectively.

Intradermal DC immunizations had little effect on distribution and activation of the three NK cell subsets (Figures 4, 5). They may have had a modest suppressive (“normalizing”) impact on CXCR1 expression levels on CD56^bright^ CD16^−^ and CD56^dim^ CD16^−^ NK cells (Figure 5; not significant). All patients tested who subsequently received HDI had increased presence of circulating CD56^bright^ CD16^−^ NK cell frequencies (Figure 6A). HDI therapy did not significantly impact expression levels of activation and chemokine receptors evaluated on any of the NK cell subsets (Figures 6B, 7).

### Cytokine and Chemokine Levels Measured in Patient Sera Prior to and Post DC and HDI Therapies

Sera were evaluated for the presence of multiple cytokines (e.g., IL-12p70, IL-18, IL-15, TNF) and chemokines (e.g., IL-8/CXCL8, IP-10/CXCL10) associated with NK cell activation and recruitment. None of the cytokines evaluated were detectable on a consistent basis in either HD or patient sera (data not shown). In contrast, measurable levels of MCP-1/CCL2, MIP-1α/CCL3, MIP-1β/CCL4, RANTES/CCL5, eotaxin-1/CCL11, IP-10/CXCL10, and SDF-1α/CXCL12 were found in the majority of donor sera tested. Patients and HDs displayed similar serum levels of all detectable chemokines with the exception of eotaxin-1/CCL11 which was slightly decreased in patient sera, particularly in patients with measurable disease (Supplemental Figure 5). DC therapy led to a slight increase in MIP-1α/CCL3 and RANTES/CCL5, while HDI had no effect on chemokine levels in patient sera (Supplemental Figure 6).

### NK Cells Isolated From Melanoma Patients With Measurable Disease Display Elevated Lytic Ability

**Figure 8:**
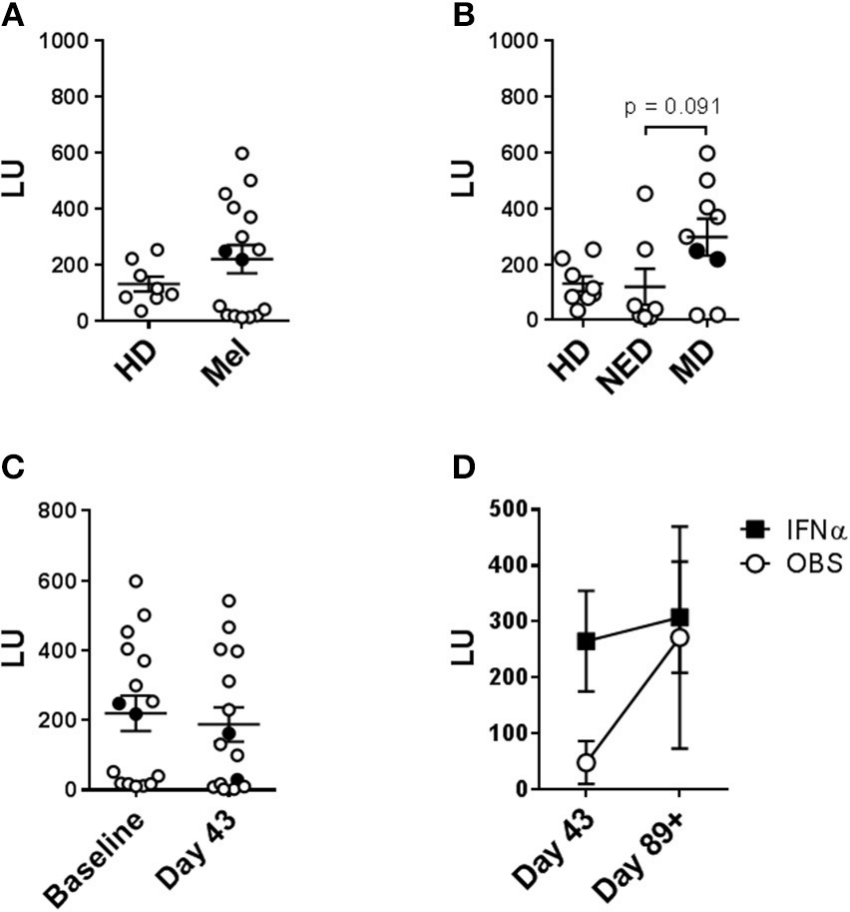
Evaluation of the impact of tumor load, DC vaccine and systemic IFNα therapies on the circulating NK cell lytic ability. Purified circulating NK cells from HD and melanoma patients (Mel) were tested by NK-TVA assay (Supplemental Figure 6) for their ability to lyse K562 targets as depicted by lytic units (LU). NK cell subsets isolated from HD and melanoma patients (Mel) was measured. **(A,B)** Melanoma patient NK cells isolated prior to treatments (Baseline) were evaluated. **(B)** NED and MD patients were compared for their NK cell subset distributions to HDs. **(C)** NK cell subset distributions prior to all the treatments (Baseline) and after DC immunizations (Day 43) are compared. **(D)** NK cell subset distributions post DC immunizations are compared for patients in the observation arm of the study and those that received HDI (IFNα). Intersecting lines and whiskers represent mean and standard error of mean values, respectively. Black circles **(A–C)** represent patients that were PR.

Lytic capability of patient peripheral blood NK cells was also examined because this metric has previously been correlated with disease incidence and prognosis (28). Patient and HD systemic NK cells were evaluated on per-cell basis. Purified NK cells were tested at different effector-to-target (E:T) ratios against K562 cells using the NK-TVA assay (Supplemental Figure 7). At baseline, melanoma patient NK cells showed elevated mean lytic ability vs. HDs (189 vs. 131 LU; Figure 8A). There were two groups of patients: those that had lower lytic ability than HDs and those that had higher. Closer evaluation of patient NK cell responses indicated that 5/7 NED patients showed decreased (120 LU on average), while 7/9 patients with measurable disease displayed increased killing (300 LU on average) ability (Figure 8B). Interestingly, the two NED patients that showed elevated killing ability (Mel13 and Mel21; Supplemental Table 1) are those that clinically progressed following the completion of the trial (Figure 8B; Mel13 and Mel 21 had progression-free survival of 18.4 and 19.2 months, respectively; Butterfield et al., under review). DC immunizations and systemic HDI therapy did not significantly impact the NK cell lytic ability significantly (Figures 8C,D).

### Enhanced NK Cell Lytic Ability Strongly Correlates With Enhanced CD56^dim^ CD16^+^ NK Cell Presence

**Figure 9:**
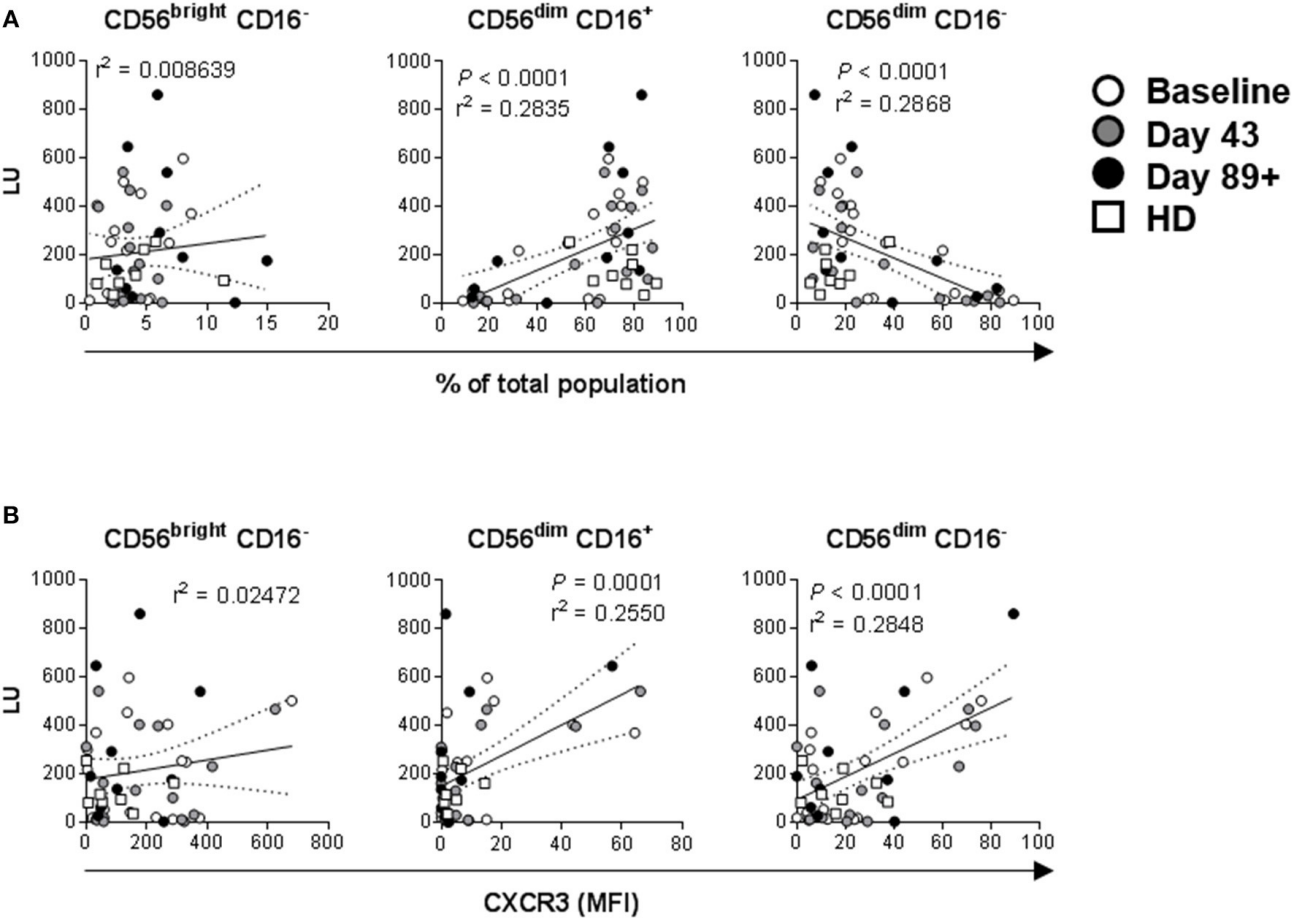
Correlations between NK cell lytic ability, distribution of the three major NK cell subsets, and expression levels of activation markers. Complementary data values acquired for each of the donors and time-points were evaluated. **(A)** Correlations between LU (Figure 7) and distribution values for each of the three major NK cell subsets based CD56 and CD16 expression levels (Figures 2A, 4A, 6A, Supplemental Figure 3A) are shown. *P*-values are shown in red color. **(B)** Correlations between LU and CXCR3 MFI levels (Figures 3, 5, 7, Supplemental Figure 4) measured on the three NK cell subsets is shown.

Lytic unit values calculated for each donor sample and collection time-point were cumulatively correlated with paired percentage distributions of each major NK cell subset. There was a highly significant positive correlation between LUs and CD56^dim^ CD16^+^ cell prevalence, as expected based on their known cytotoxic phenotype (Figure 9A) (20, 44–47). Reciprocally, there was a significant negative correlation between LU and CD56^dim^ CD16^−^ cells, whereas there was no correlation for CD56^bright^ CD16^−^ cell percentage (Figure 9A). In agreement, CD56^dim^ CD16^+^ NK cell percentage values inversely correlated to paired CD56^dim^ CD16^−^ percentages (Supplemental Figure 8). These data indicate that CD56^dim^ CD16^−^ cells likely have a different function. The relationship between LU and expression levels of tested markers was explored and expression of CXCR3 on CD56^dim^ CD16^+^ and, surprisingly, CD56^dim^ CD16^−^ positively correlates with LU (Figure 9B). Increased CD56^dim^ CD16^−^ NK cell incidence did not correlate with NKp30, NKp46, CXCR1 and CCR7 expression levels (data not shown). However, enhanced expression levels of CD69 and CXCR3 did positively and negatively correlate with CD56^dim^ CD16^−^ NK cell presence, respectively (Supplemental Figure 9), suggesting that elevated CD56^dim^ CD16^−^ NK cell presence in some patients may be due to their dysregulated retention in circulation because of altered expression levels of CD69 (48) and CXCR3.

### CD56^dim^ CD16^−^ NK Cells Are the Dominant Subset in the Melanoma Microenvironment

**Figure 10:**
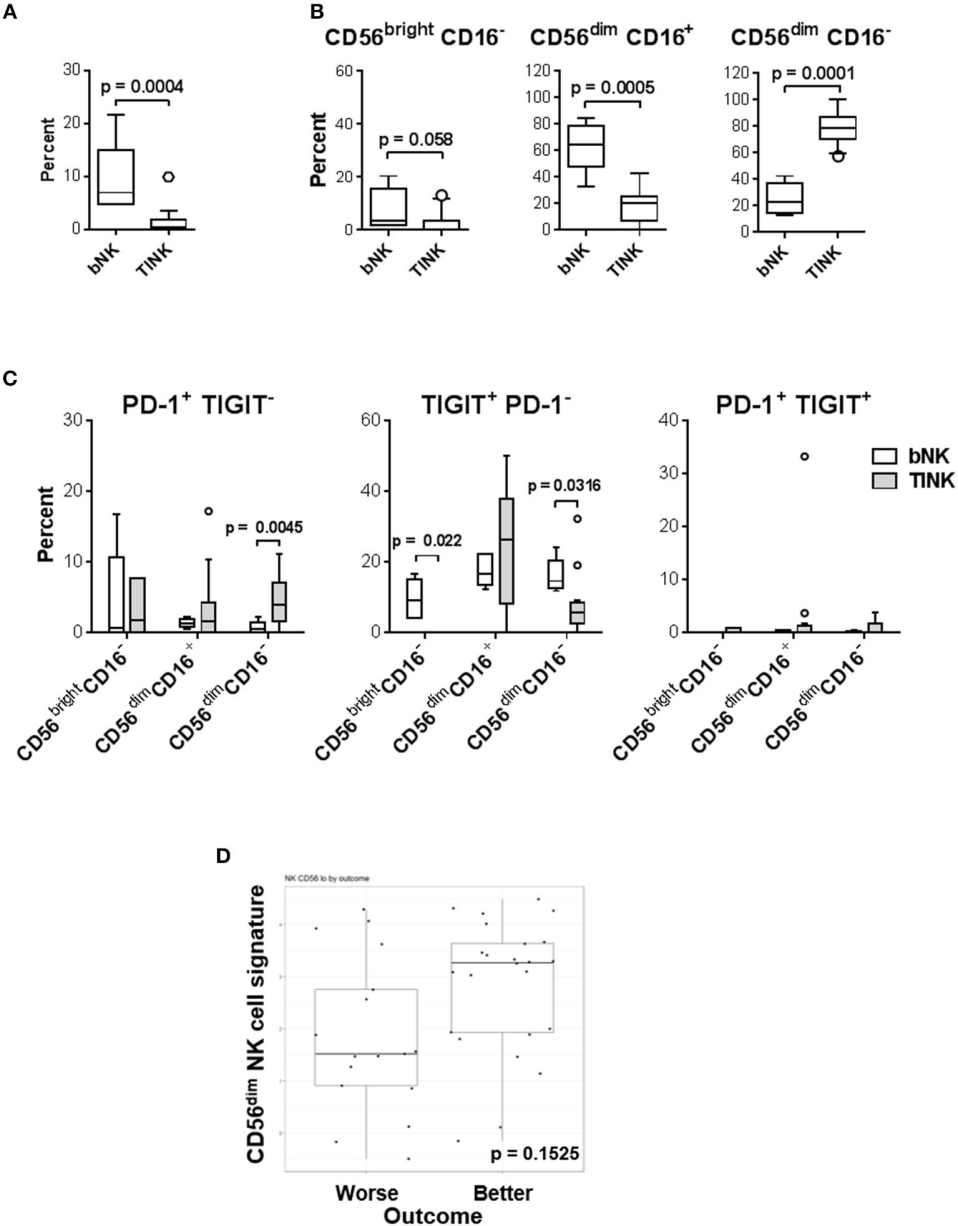
CD56^dim^ CD16^−^ NK cells are the most abundant TINKs in melanoma biopsies. **(A–C)** PBMC (*n* = 5) and bulk single-cell tumor suspensions (*n* = 15) were isolated from melanoma patients not enrolled in the trial (Supplemental Table 2) and were evaluated by multi-color flow cytometry for T cell (CD3^+^) and NK cell subset distribution patterns based on CD56 and CD16 expression levels. Gating strategy is shown in Supplemental Figure 10. **(A)** Percent distribution of the total NK cell population in blood (PBMC) and tumor (TILs) is shown. **(B)** Distribution of the three major NK cell subsets among blood (bNK) and TINK is shown. **(C)** Expression patterns of PD-1 and TIGIT on the three NK cell subsets in blood and tumors are shown. **(B,C)** Data are presented using Tukey's box plot analysis, where intersecting lines and whiskers represent median percent and data distribution values, respectively. Circles represent data outliers. **(D)** 41 bulk single-cell tumor suspensions isolated from 19 melanoma patients enrolled in the clinical trial were tested using the NanoString nCounter PanCancer Immune Profiling Panel. CD56^dim^ gene signature was determined by IL21R, KIR2DL3, KIR3DL1, and KIR3DL2 expression patterns.

Since the two circulating CD56^dim^ subsets appear to be dysregulated in a cohort of melanoma patients and mediate distinct functions it was important to evaluate which of these subsets infiltrates melanoma. As viable tumor biopsies from the patients enrolled in our clinical trial were limited, we analyzed PBMC and melanoma cells from patients not associated with the trial by multi-color flow cytometry (Supplemental Table 2, Supplemental Figures 10, 11, and Figures 10A–C). CD56^+^ CD3^−^ NK cells were found in higher frequencies among circulating vs. tumor-infiltrating leukocytes (mean 9.37 vs. 0.63% among PBMC and TIL, respectively; Figure 10A). The CD56^dim^ CD16^+^ subset was the dominant population among circulating NK cells (mean 63.32 vs. 10.474% in blood NK cells and TINK, respectively). In sharp contrast, CD56^dim^ CD16^−^ subset was the dominant population among TINK (mean 24.9 vs. 86% in blood NK cells and TINK, respectively). Interestingly, CD56^bright^ CD16^−^ subset was not detected in most of the melanoma lesions evaluated (Figure 10B). All of the gated TINK subsets expressed NKG2D along with moderate levels of NKp46, which confirmed their NK cell lineage (Supplemental Figure 10B). Expression of CD69, a marker of activation and tissue residency, was increased on all the TINK subsets (48). In contrast, expression of ANK-1, a marker expressed on CD56^dim^ CD16^−^ adherent NK cell precursors [pre-A-NK; 49] was, on average, 3-fold decreased in tumors vs. blood (Supplemental Figures 10C, 11). We also explored whether PD-1 and TIGIT were co-expressed on CD56^dim^ CD16^−^ TINKs as a surrogate measure for functional exhaustion (49). Unlike tumor-infiltrating T cells (Supplemental Figure 10D), almost no co-expression of PD-1 and TIGIT was observed on any of the TINK subsets (Figure 10C). Interestingly, PD-1 expression was elevated on a cohort of CD56^dim^ CD16^−^ TINKs (mean 0.7 vs. 4.5% in blood NK cells and TINK, respectively), while TIGIT was downregulated on a separate cohort of CD56^dim^ CD16^−^ (mean 16.2 vs. 8.1% in blood NK cells and TINK, respectively), as well as on CD56^bright^ CD16^−^ TINKs (mean 9.5 vs. 0.5% in blood NK cells and TINK, respectively). CD56^dim^ CD16^+^ NK cells consistently presented highest levels of TIGIT, with TINKs displaying increased average expression levels (mean 17.8 vs. 24.6% in blood NK cells and TINK, respectively), however this observation did not achieve statistical significance.

### CD56^dim^ NK Cell Gene Signature in Melanomas Is Higher in Patients With Better Clinical Response

To relate aforementioned TINK observations to patients enrolled in our clinical trial, total RNA samples isolated from 16 patient biopsies prior to all treatments were evaluated by NanoString. Relative cell population abundance was evaluated as previously described (50). The CD56^dim^ NK cell gene signature was higher in patients with better clinical outcome (PR, SD and NED; Figure 10D), but the trend did not achieve statistical significance (*p* = 0.1525). Cumulatively, these data suggest that increased melanoma infiltration by CD56^dim^ CD16^−^ NK cells may be beneficial to patient outcome.

## Discussion

Our group has performed a phase I trial where a novel, type 1-skewing AdV.DC vaccine was used to promote T cell responses against tyrosinase, MART-1 and MAGE-A6. Twenty-four patients with measurable disease and 11 surgically treated NED patients were enrolled. Two out of Twenty-Four patients with measurable disease achieved a PR and 8/24 patients had stabilization of the disease. Of 11 NED patients, 4 remain NED at a median follow-up of 3 years (Butterfield et al., under review). While the vaccine induced T cell responses against multiple antigens, it was also important to characterize whether AdV.DC ± HDI affected peripheral blood NK cells as predicted by our pre-clinical studies in terms of subset distribution, activation and chemokine receptor expression, and functionality.

NK cells have traditionally been studied as predominantly two separate populations, CD56^bright^ CD16^−^ and CD56^dim^ CD16^+^, more attention has recently been given to the CD56^dim^ CD16^−^ subset. CD56^bright^ CD16^−^cells exhibit superior cytokine production, whereas CD56^dim^ CD16^+^ cells primarily demonstrate enhanced cytotoxicity (20, 44–47). CD56^bright^ CD16^−^ NK cells preferentially localize to secondary lymphoid tissues and CD56^dim^ CD16^+^ cells occupy peripheral blood, lungs, and sites of inflammation (45, 51–54). Such tropisms are determined by unique chemokine receptor expression profiles which include CCR7, and CXCR3 for CD56^bright^ CD16^−^ cells and CXCR1 and CX_3_CR1 for CD56^dim^ CD16^+^ NK cells (33, 53, 55).

The CD56^dim^ CD16^−^ NK subset may be a highly heterogenous population consisting of both maturing and target cell-activated cells. Precursors of adherent NK cells (pre-A-NK) that express ANK-1, a polysialylated 230-kD isoform of NCAM, have been identified within the CD56^dim^ CD16^−^ NK cell population (56). We show that ANK-1^+^ CD56^dim^ CD16^−^ NK cells are underrepresented within melanoma lesions as compared to circulation, likely indicating altered cytolytic state of CD56^dim^ CD16^−^ TINKs. More recent studies have shown that there are both poorly and highly cytotoxic populations found within this subset (57, 58). In concert with these reports, CD16 expression on CD56^dim^ CD16^+^ cells has been shown to be downregulated following target cell-induced activation of matrix metalloproteases, specifically ADAM17. CD16 shedding strongly correlates with increased CD107a expression, indicating that degranulation coincides with CD16 downregulation (59, 60).

Enhanced understanding of NK responses as well as the mechanisms by which NK cells promote immunity in these therapies could benefit patient outcomes by: (1) identifying suitable treatment candidates based on NK cell distributions and marker expression levels; (2) provide a system to evaluate patient disease progression or regression in response to treatment; (3) determine the potential efficacy of IFN-α adjuvant for specific patients. Though it was expected that NK cells from patients with measurable disease would show decreased expression of activating receptors and extravasation-associated receptors, as well as lower lytic ability (61), the opposite was found. While a number of soluble immunosuppressive factors were found to be elevated in the sera of metastatic disease patients, their NK cells displayed increased expression of multiple activation markers and chemokine receptors, and enhanced lytic ability that directly correlated with higher representation of CD56^dim^ CD16^+^ NK cells. A previous study has shown that CD56^dim^ CD57^lo^ CD69^+^ CCR7^+^ KIR^+^ NK cells expand in tumor-infiltrated lymph nodes and display enhanced cytotoxic activity against autologous melanoma cells. The same study has shown that the frequency of circulating NK cells expressing the receptors for IL-8/CXCL8 is increased in metastatic melanoma patients compared with healthy subjects, agreeing with our results (32). The observation that systemic NK cells in patients with metastatic melanoma display an enhanced degree of activation may be the consequence of lack of extravasation by activated NK cells into tumors, thereby increasing the presence of systemic NK cells with elevated lytic ability. It has been shown that stage I melanoma patients with high systemic NK cell activity have less lymphocyte infiltrate at the base of the tumor than those with low NK cell activity (62). To address this hypothesis, tumor infiltration will have to be analyzed for activated NK cells. Our exploratory transcriptomic analysis indicates that the CD56^dim^ NK cell gene signature in bulk tumor biopsies is associated with better clinical response. Phenotypic evaluation of TINKs suggests that the CD56^dim^ CD16^−^ subset is the dominant NK cell population in melanomas. These results are not surprising as we have previously shown that the same subset in blood expresses the broadest repertoire of chemokine receptors, including CCR2, CCR3, CCR4, and CCR5 which are not expressed on other subsets, indicating that these NK cells are highly capable of recruitment into inflamed tissues (33). We also show that TINKs likely do not have the commonly-used “exhaustion” (co-expression of multiple checkpoints, like PD-1 and TIGIT). Future studies need to explore in greater detail the function and functional state of tumor-infiltrating CD56^dim^ CD16^−^ NK cells subsets in blood and in the tumor microenvironment. Based on our cumulative data, as well as reports that TINKs are not as cytotoxic as their blood counterparts (63), we hypothesize that this subset is likely not cytotoxic, that it lacks the ability to perform antibody-dependent cell-mediated cytotoxicity due to lack of CD16 expression and that its major anti-tumor activity is likely mediated by secreted factors such as cytokines, chemokines, and growth factors. Additionally, the number of CD56^bright^ TINKs may be underestimated due to usage of an anti-CD56 antibody that is conjugated to a moderately bright fluorochrome (BV510). Consequently, future studies also need to re-confirm our observations using anti-CD56 antibodies conjugated to brighter fluorochromes (e.g., PE or BV421).

Intradermal AdV.DC treatments did not appear to have a significant effect on NK cell phenotype and function. There are three possible reasons for this: (1) the time between DC injection and blood draw led to the failure to detect AdV.DC impact; (2) intradermal AdV.DC immunizations are not the optimal route of administration to induce systemic NK cell activation; and (3) the AdV.DC vaccine as formulated was incapable of proper recruitment and activation of NK cells. One month of systemic HDI administration following AdV.DC immunizations, which has been shown to boost adaptive immune responses and NK cell functionality (12–15), appears to promote replenishment of naïve NK cells in circulation in these patients as measured by increased representation of CD56^bright^ CD16^−^ NK cells. There appears to be a role for CD56^dim^ CD16^+^ and CD56^dim^ CD16^−^ NK cells in response to tumors. CD56^dim^ CD16^−^ NK cell prevalence negatively correlated with lytic ability, and appeared to inversely correlate with the presence of cytotoxic CD56^dim^ CD16^+^ NK cells. The developmental stage of this subset, as well the as biological function, is equivocal. The prevalence of this subset in the tumor may indicate aberrant cytotoxic capability. It may also indicate that patient samples contain mostly partially differentiated NK cells as CD56^dim^ KIR^−^ CD16^−^ perforin^+^ cells have been reported to be an intermediate stage NK cell differentiation (64). Functional analyses of these cells isolated *ex vivo* would confirm the phenotype.

In summary, we performed a detailed phenotypic and functional analysis of blood NK cells isolated from melanoma patients treated with AdV.DC ± HDI. We show that melanoma patient NK cells display elevated activation levels and that CD56^dim^ CD16^−^ NK cells are a unique non-cytolytic subset in melanoma patients that may positively impact patients' clinical outcome.

## Ethics Statement

This study was carried out in accordance with the recommendations of the University of Pittsburgh Institutional Review Board (IRB) with written informed consent from all subjects (HCC protocols 04-001, 09-021 and 96-099). All subjects gave written informed consent in accordance with the Declaration of Helsinki. The protocol was approved by the University of Pittsburgh IRB.

## Author Contributions

LV and LB were accountable for the conception and design of research and writing the manuscript. LV performed most, while CC, PS, and JL performed some of *in vitro* experiments and data analysis. YL and FD performed all biostatistical analyses. JK was the clinical trial leader. CS processed and coordinated tumor biopsies. AW and AH processed samples for NanoString testing. AM-H and SW analyzed NanoString data.

### Conflict of Interest Statement

The authors declare that the research was conducted in the absence of any commercial or financial relationships that could be construed as a potential conflict of interest.
