# Supplementary material for: CD56dim CD16− Natural Killer Cell Profiling in Melanoma Patients Receiving a Cancer Vaccine and Interferon-α
Source: Front Immunol. 2019 Jan 29;10:14. doi: 10.3389/fimmu.2019.00014 (PMC6361792; doi:10.3389/fimmu.2019.00014)
Supplement: Supplementary file 1 [file Presentation_1.pptx]

## Slide 1
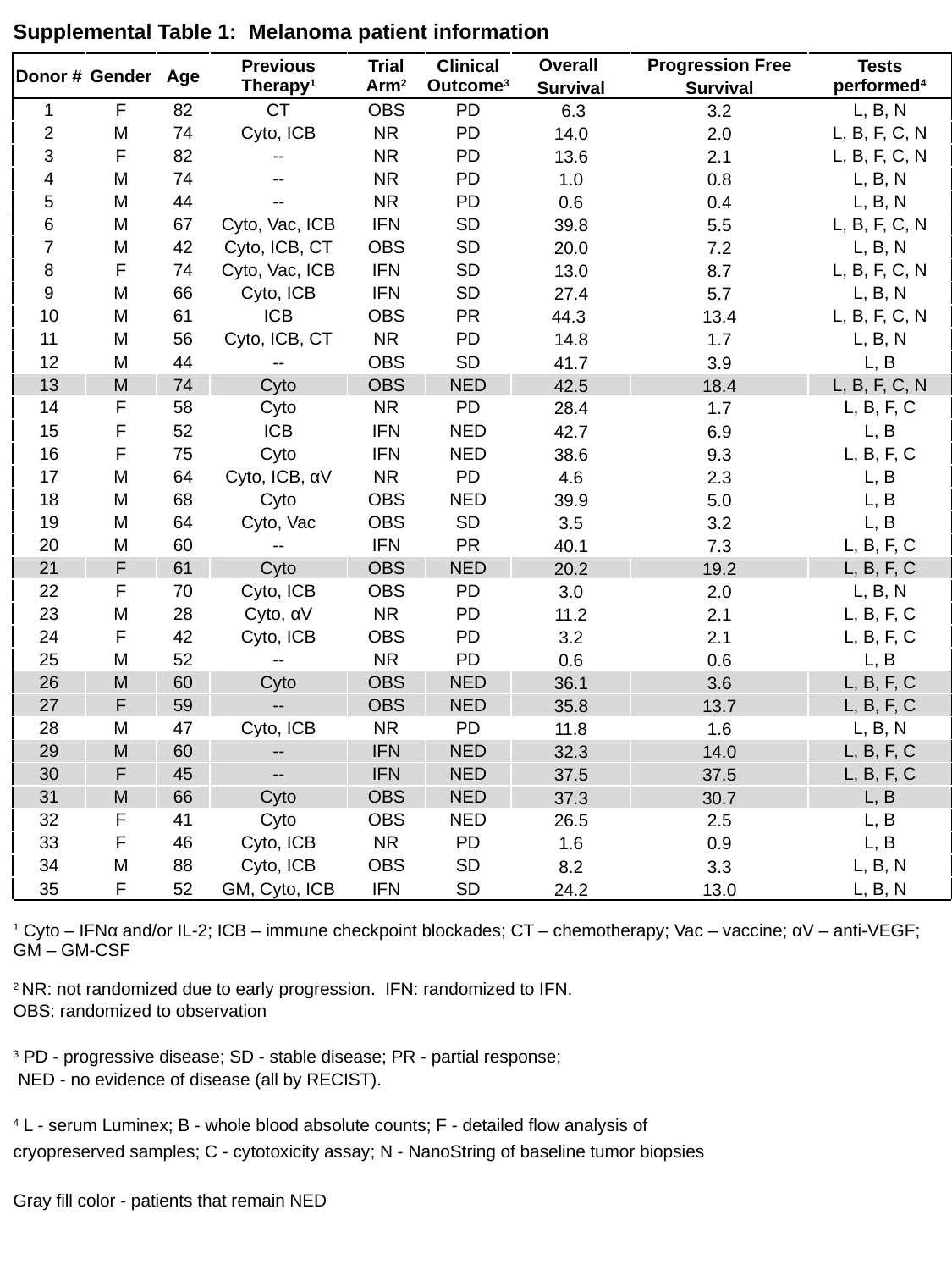

| Supplemental Table 1:  Melanoma patient information | | | | | | | | | |
| --- | --- | --- | --- | --- | --- | --- | --- | --- | --- |
| Donor # | Gender | Age | Previous Therapy1 | Trial Arm2 | Clinical Outcome3 | Overall Survival | Progression Free Survival | Progression Free Survival | Tests performed4 |
| 1 | F | 82 | CT | OBS | PD | 6.3 | 3.2 | 3.2 | L, B, N |
| 2 | M | 74 | Cyto, ICB | NR | PD | 14.0 | 2.0 | 2.0 | L, B, F, C, N |
| 3 | F | 82 | -- | NR | PD | 13.6 | 2.1 | 2.1 | L, B, F, C, N |
| 4 | M | 74 | -- | NR | PD | 1.0 | 0.8 | 0.8 | L, B, N |
| 5 | M | 44 | -- | NR | PD | 0.6 | 0.4 | 0.4 | L, B, N |
| 6 | M | 67 | Cyto, Vac, ICB | IFN | SD | 39.8 | 5.5 | 5.5 | L, B, F, C, N |
| 7 | M | 42 | Cyto, ICB, CT | OBS | SD | 20.0 | 7.2 | 7.2 | L, B, N |
| 8 | F | 74 | Cyto, Vac, ICB | IFN | SD | 13.0 | 8.7 | 8.7 | L, B, F, C, N |
| 9 | M | 66 | Cyto, ICB | IFN | SD | 27.4 | 5.7 | 5.7 | L, B, N |
| 10 | M | 61 | ICB | OBS | PR | 44.3 | 13.4 | 13.4 | L, B, F, C, N |
| 11 | M | 56 | Cyto, ICB, CT | NR | PD | 14.8 | 1.7 | 1.7 | L, B, N |
| 12 | M | 44 | -- | OBS | SD | 41.7 | 3.9 | 3.9 | L, B |
| 13 | M | 74 | Cyto | OBS | NED | 42.5 | 18.4 | 18.4 | L, B, F, C, N |
| 14 | F | 58 | Cyto | NR | PD | 28.4 | 1.7 | 1.7 | L, B, F, C |
| 15 | F | 52 | ICB | IFN | NED | 42.7 | 6.9 | 6.9 | L, B |
| 16 | F | 75 | Cyto | IFN | NED | 38.6 | 9.3 | 9.3 | L, B, F, C |
| 17 | M | 64 | Cyto, ICB, αV | NR | PD | 4.6 | 2.3 | 2.3 | L, B |
| 18 | M | 68 | Cyto | OBS | NED | 39.9 | 5.0 | 5.0 | L, B |
| 19 | M | 64 | Cyto, Vac | OBS | SD | 3.5 | 3.2 | 3.2 | L, B |
| 20 | M | 60 | -- | IFN | PR | 40.1 | 7.3 | 7.3 | L, B, F, C |
| 21 | F | 61 | Cyto | OBS | NED | 20.2 | 19.2 | 19.2 | L, B, F, C |
| 22 | F | 70 | Cyto, ICB | OBS | PD | 3.0 | 2.0 | 2.0 | L, B, N |
| 23 | M | 28 | Cyto, αV | NR | PD | 11.2 | 2.1 | 2.1 | L, B, F, C |
| 24 | F | 42 | Cyto, ICB | OBS | PD | 3.2 | 2.1 | 2.1 | L, B, F, C |
| 25 | M | 52 | -- | NR | PD | 0.6 | 0.6 | 0.6 | L, B |
| 26 | M | 60 | Cyto | OBS | NED | 36.1 | 3.6 | 3.6 | L, B, F, C |
| 27 | F | 59 | -- | OBS | NED | 35.8 | 13.7 | 13.7 | L, B, F, C |
| 28 | M | 47 | Cyto, ICB | NR | PD | 11.8 | 1.6 | 1.6 | L, B, N |
| 29 | M | 60 | -- | IFN | NED | 32.3 | 14.0 | 14.0 | L, B, F, C |
| 30 | F | 45 | -- | IFN | NED | 37.5 | 37.5 | 37.5 | L, B, F, C |
| 31 | M | 66 | Cyto | OBS | NED | 37.3 | 30.7 | 30.7 | L, B |
| 32 | F | 41 | Cyto | OBS | NED | 26.5 | 2.5 | 2.5 | L, B |
| 33 | F | 46 | Cyto, ICB | NR | PD | 1.6 | 0.9 | 0.9 | L, B |
| 34 | M | 88 | Cyto, ICB | OBS | SD | 8.2 | 3.3 | 3.3 | L, B, N |
| 35 | F | 52 | GM, Cyto, ICB | IFN | SD | 24.2 | 13.0 | 13.0 | L, B, N |
| 1 Cyto – IFNα and/or IL-2; ICB – immune checkpoint blockades; CT – chemotherapy; Vac – vaccine; αV – anti-VEGF; GM – GM-CSF 2 NR: not randomized due to early progression.  IFN: randomized to IFN. | | | | | | | | | |
| OBS: randomized to observation | | | | | | | | | |
| | | | | | | | | | |
| 3 PD - progressive disease; SD - stable disease; PR - partial response; | | | | | | | | | |
| NED - no evidence of disease (all by RECIST). | | | | | | | | | |
| | | | | | | | | | |
| 4 L - serum Luminex; B - whole blood absolute counts; F - detailed flow analysis of | | | | | | | | | |
| cryopreserved samples; C - cytotoxicity assay; N - NanoString of baseline tumor biopsies | | | | | | | | | |
| | | | | | | | | | |
| Gray fill color - patients that remain NED | | | | | | | | | |

## Slide 2
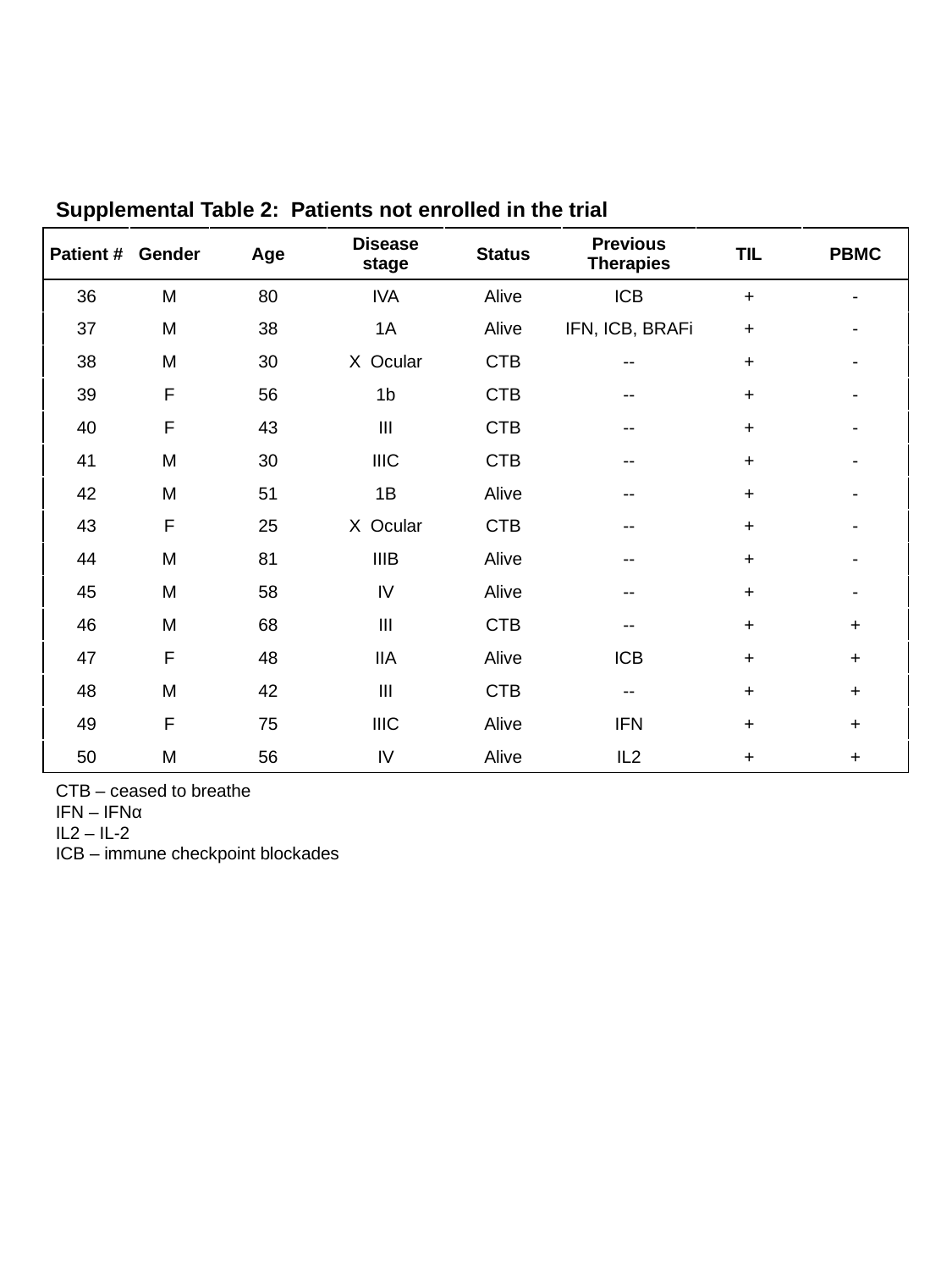

Supplemental Table 2:  Patients not enrolled in the trial
| Patient # | Gender | Age | Disease stage | Status | Previous Therapies | TIL | PBMC |
| --- | --- | --- | --- | --- | --- | --- | --- |
| 36 | M | 80 | IVA | Alive | ICB | + | - |
| 37 | M | 38 | 1A | Alive | IFN, ICB, BRAFi | + | - |
| 38 | M | 30 | X Ocular | CTB | -- | + | - |
| 39 | F | 56 | 1b | CTB | -- | + | - |
| 40 | F | 43 | III | CTB | -- | + | - |
| 41 | M | 30 | IIIC | CTB | -- | + | - |
| 42 | M | 51 | 1B | Alive | -- | + | - |
| 43 | F | 25 | X Ocular | CTB | -- | + | - |
| 44 | M | 81 | IIIB | Alive | -- | + | - |
| 45 | M | 58 | IV | Alive | -- | + | - |
| 46 | M | 68 | III | CTB | -- | + | + |
| 47 | F | 48 | IIA | Alive | ICB | + | + |
| 48 | M | 42 | III | CTB | -- | + | + |
| 49 | F | 75 | IIIC | Alive | IFN | + | + |
| 50 | M | 56 | IV | Alive | IL2 | + | + |
CTB – ceased to breathe
IFN – IFNα
IL2 – IL-2
ICB – immune checkpoint blockades

## Slide 3
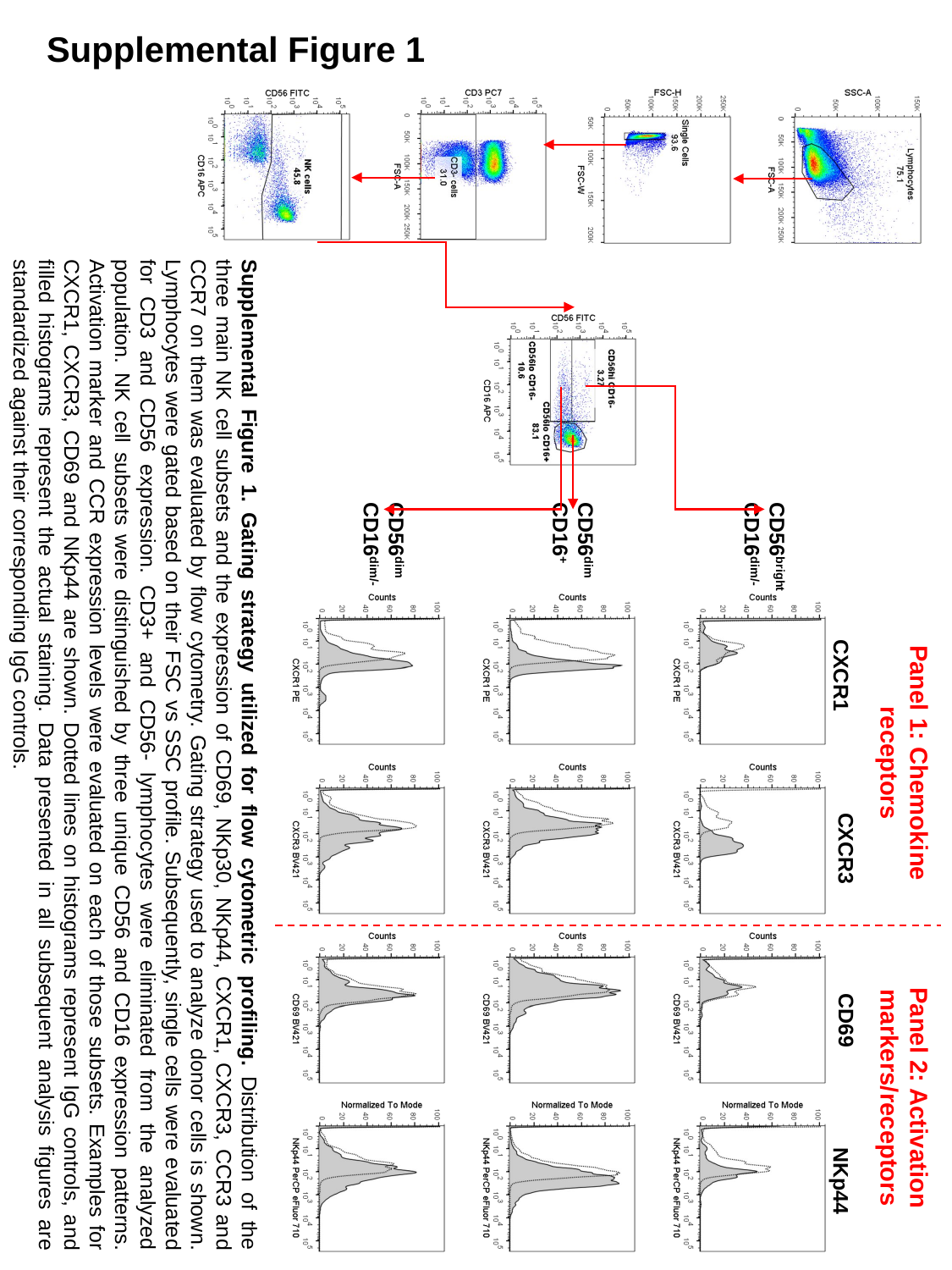

Supplemental Figure 1
CD56dim CD16dim/-
CD56dim CD16+
CD56bright CD16dim/-
CXCR3
Supplemental Figure 1. Gating strategy utilized for flow cytometric profiling. Distribution of the three main NK cell subsets and the expression of CD69, NKp30, NKp44, CXCR1, CXCR3, CCR3 and CCR7 on them was evaluated by flow cytometry. Gating strategy used to analyze donor cells is shown. Lymphocytes were gated based on their FSC vs SSC profile. Subsequently, single cells were evaluated for CD3 and CD56 expression. CD3+ and CD56- lymphocytes were eliminated from the analyzed population. NK cell subsets were distinguished by three unique CD56 and CD16 expression patterns. Activation marker and CCR expression levels were evaluated on each of those subsets. Examples for CXCR1, CXCR3, CD69 and NKp44 are shown. Dotted lines on histograms represent IgG controls, and filled histograms represent the actual staining. Data presented in all subsequent analysis figures are standardized against their corresponding IgG controls.
CXCR1
CD69
Panel 1: Chemokine receptors
NKp44
Panel 2: Activation markers/receptors

## Slide 4
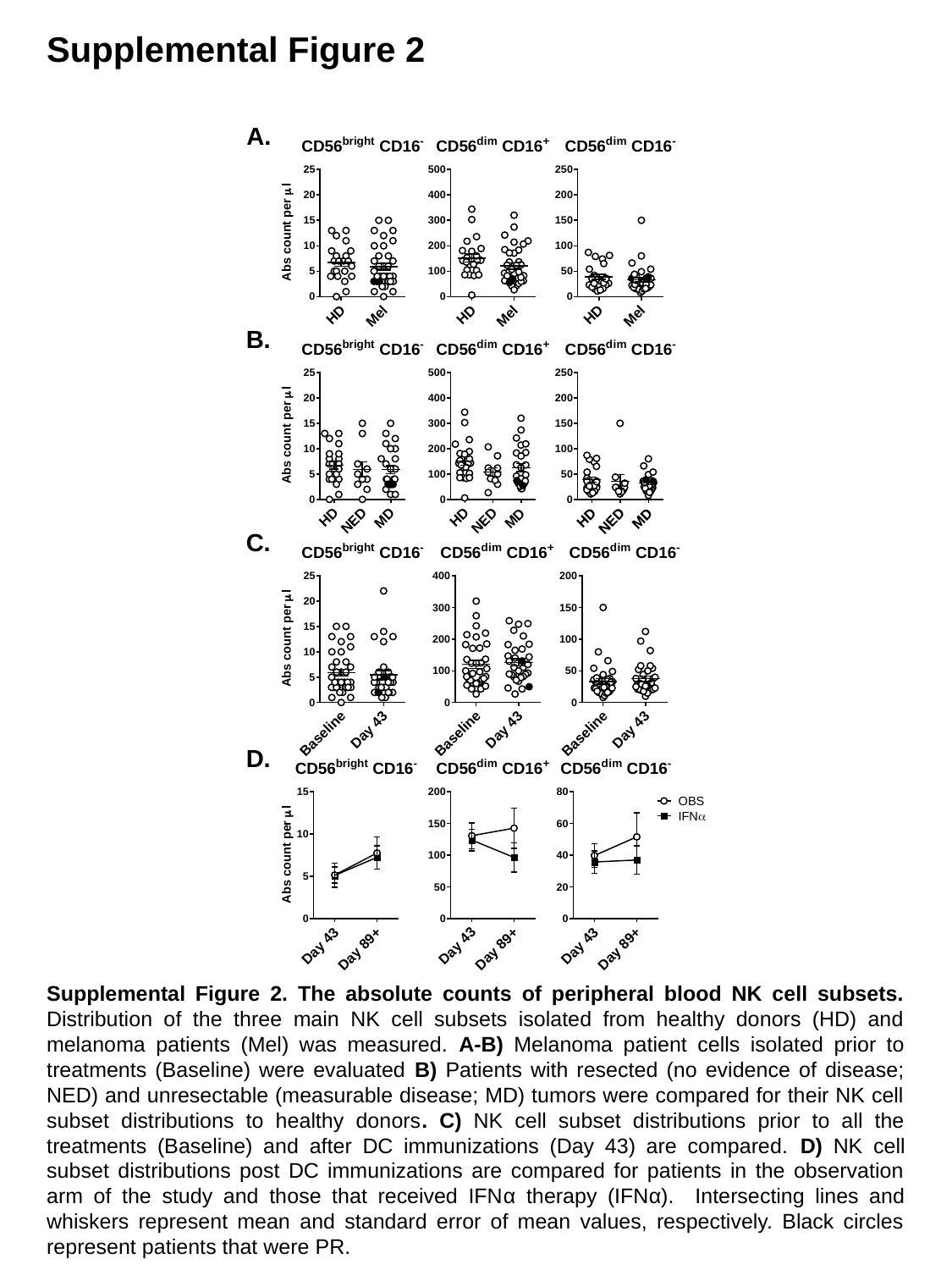

Supplemental Figure 2
A.
B.
C.
D.
Supplemental Figure 2. The absolute counts of peripheral blood NK cell subsets. Distribution of the three main NK cell subsets isolated from healthy donors (HD) and melanoma patients (Mel) was measured. A-B) Melanoma patient cells isolated prior to treatments (Baseline) were evaluated B) Patients with resected (no evidence of disease; NED) and unresectable (measurable disease; MD) tumors were compared for their NK cell subset distributions to healthy donors. C) NK cell subset distributions prior to all the treatments (Baseline) and after DC immunizations (Day 43) are compared. D) NK cell subset distributions post DC immunizations are compared for patients in the observation arm of the study and those that received IFNα therapy (IFNα). Intersecting lines and whiskers represent mean and standard error of mean values, respectively. Black circles represent patients that were PR.

## Slide 5
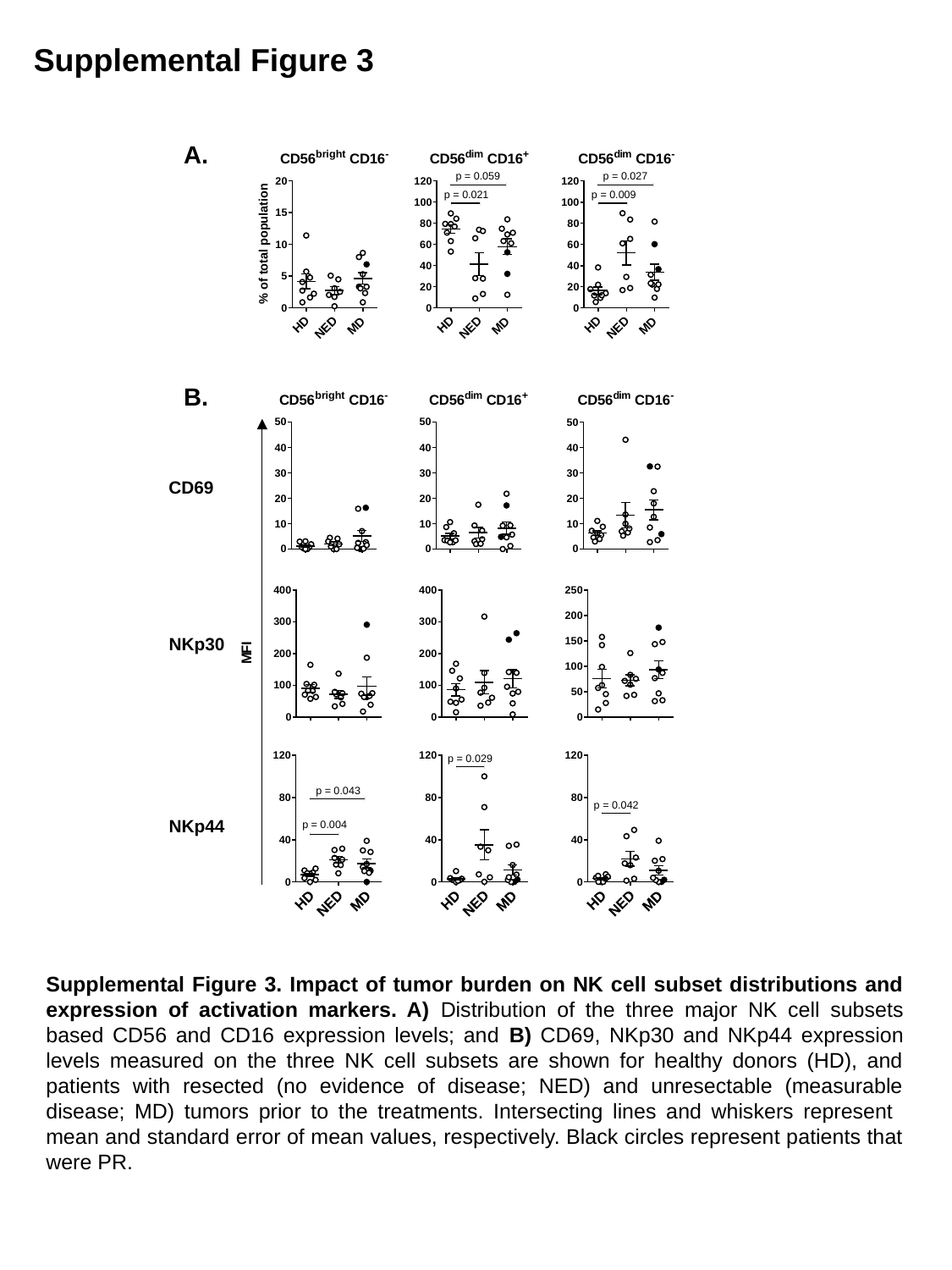

Supplemental Figure 3
A.
B.
CD69
NKp30
NKp44
Supplemental Figure 3. Impact of tumor burden on NK cell subset distributions and expression of activation markers. A) Distribution of the three major NK cell subsets based CD56 and CD16 expression levels; and B) CD69, NKp30 and NKp44 expression levels measured on the three NK cell subsets are shown for healthy donors (HD), and patients with resected (no evidence of disease; NED) and unresectable (measurable disease; MD) tumors prior to the treatments. Intersecting lines and whiskers represent mean and standard error of mean values, respectively. Black circles represent patients that were PR.

## Slide 6
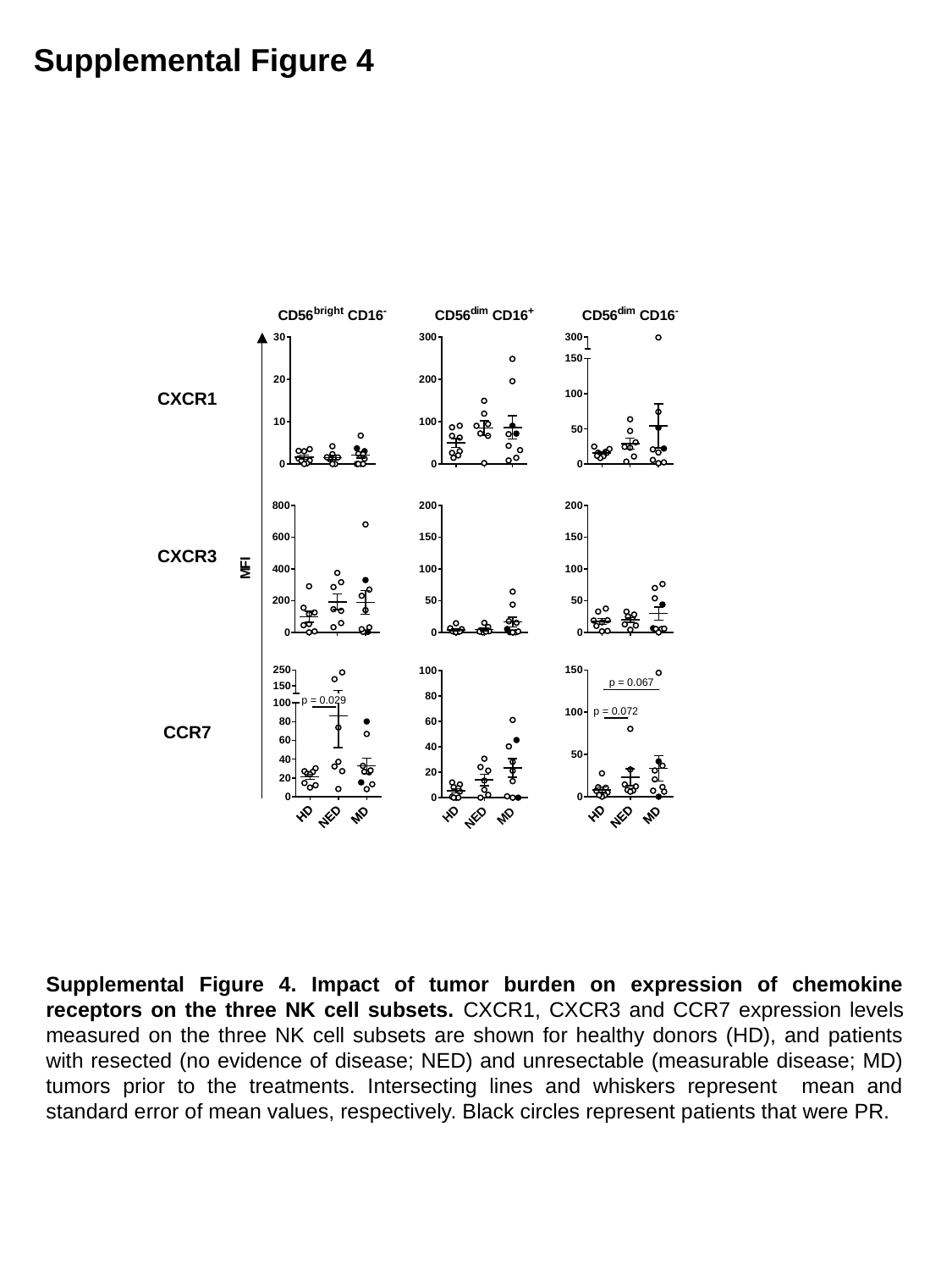

Supplemental Figure 4
CXCR1
CXCR3
CCR7
Supplemental Figure 4. Impact of tumor burden on expression of chemokine receptors on the three NK cell subsets. CXCR1, CXCR3 and CCR7 expression levels measured on the three NK cell subsets are shown for healthy donors (HD), and patients with resected (no evidence of disease; NED) and unresectable (measurable disease; MD) tumors prior to the treatments. Intersecting lines and whiskers represent mean and standard error of mean values, respectively. Black circles represent patients that were PR.

## Slide 7
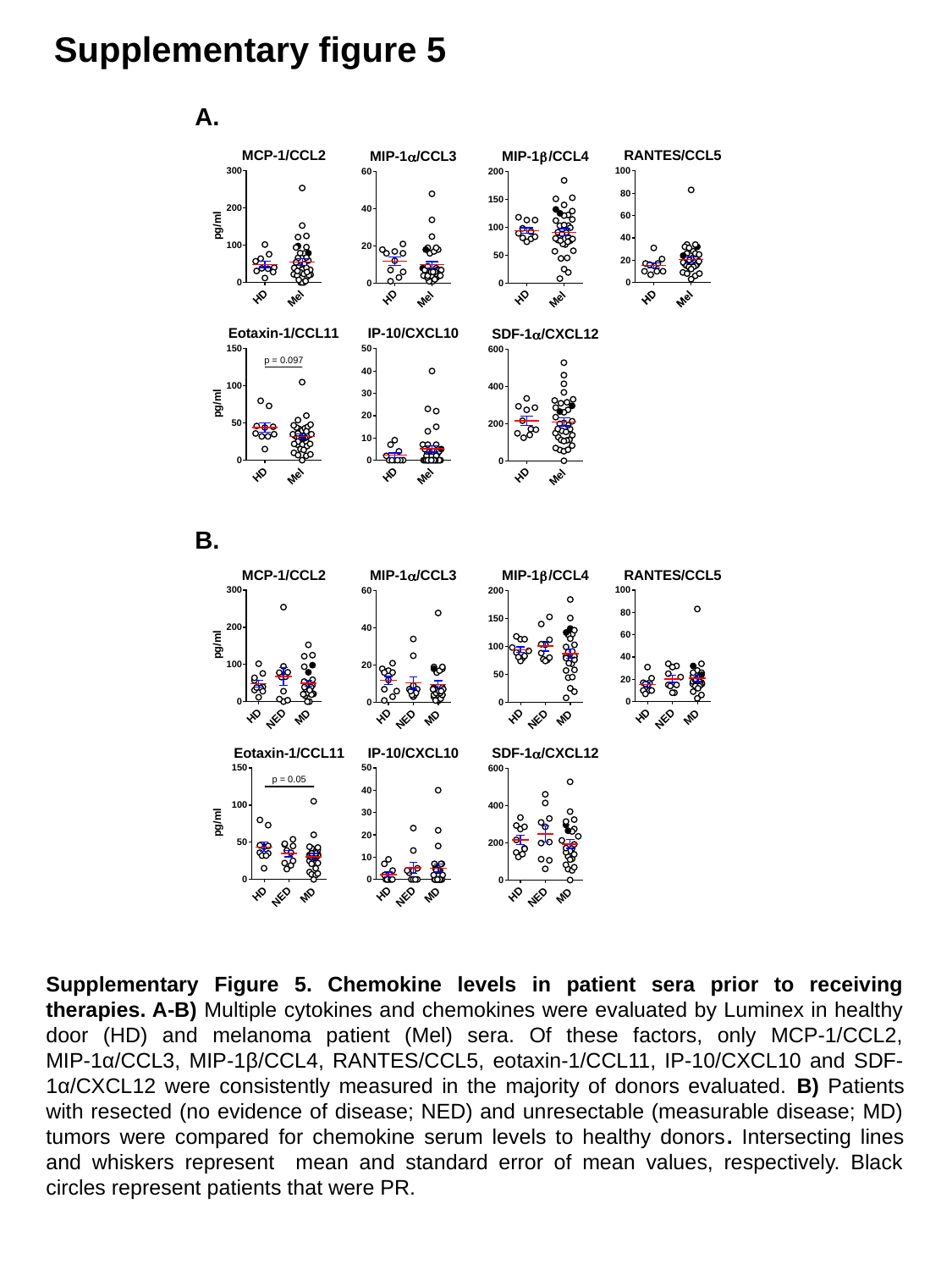

Supplementary figure 5
A.
B.
Supplementary Figure 5. Chemokine levels in patient sera prior to receiving therapies. A-B) Multiple cytokines and chemokines were evaluated by Luminex in healthy door (HD) and melanoma patient (Mel) sera. Of these factors, only MCP-1/CCL2, MIP-1α/CCL3, MIP-1β/CCL4, RANTES/CCL5, eotaxin-1/CCL11, IP-10/CXCL10 and SDF-1α/CXCL12 were consistently measured in the majority of donors evaluated. B) Patients with resected (no evidence of disease; NED) and unresectable (measurable disease; MD) tumors were compared for chemokine serum levels to healthy donors. Intersecting lines and whiskers represent mean and standard error of mean values, respectively. Black circles represent patients that were PR.

## Slide 8
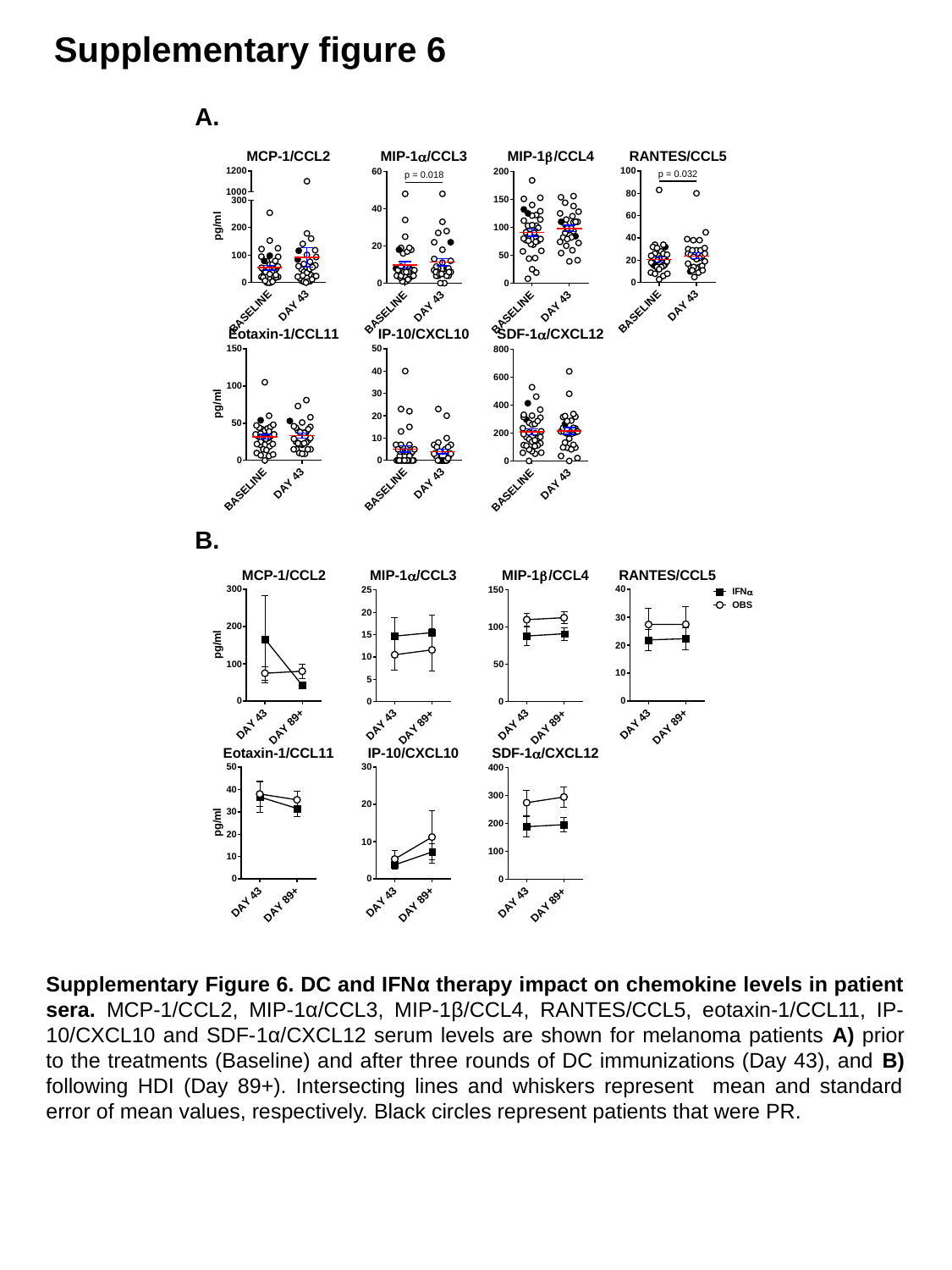

Supplementary figure 6
A.
B.
Supplementary Figure 6. DC and IFNα therapy impact on chemokine levels in patient sera. MCP-1/CCL2, MIP-1α/CCL3, MIP-1β/CCL4, RANTES/CCL5, eotaxin-1/CCL11, IP-10/CXCL10 and SDF-1α/CXCL12 serum levels are shown for melanoma patients A) prior to the treatments (Baseline) and after three rounds of DC immunizations (Day 43), and B) following HDI (Day 89+). Intersecting lines and whiskers represent mean and standard error of mean values, respectively. Black circles represent patients that were PR.

## Slide 9
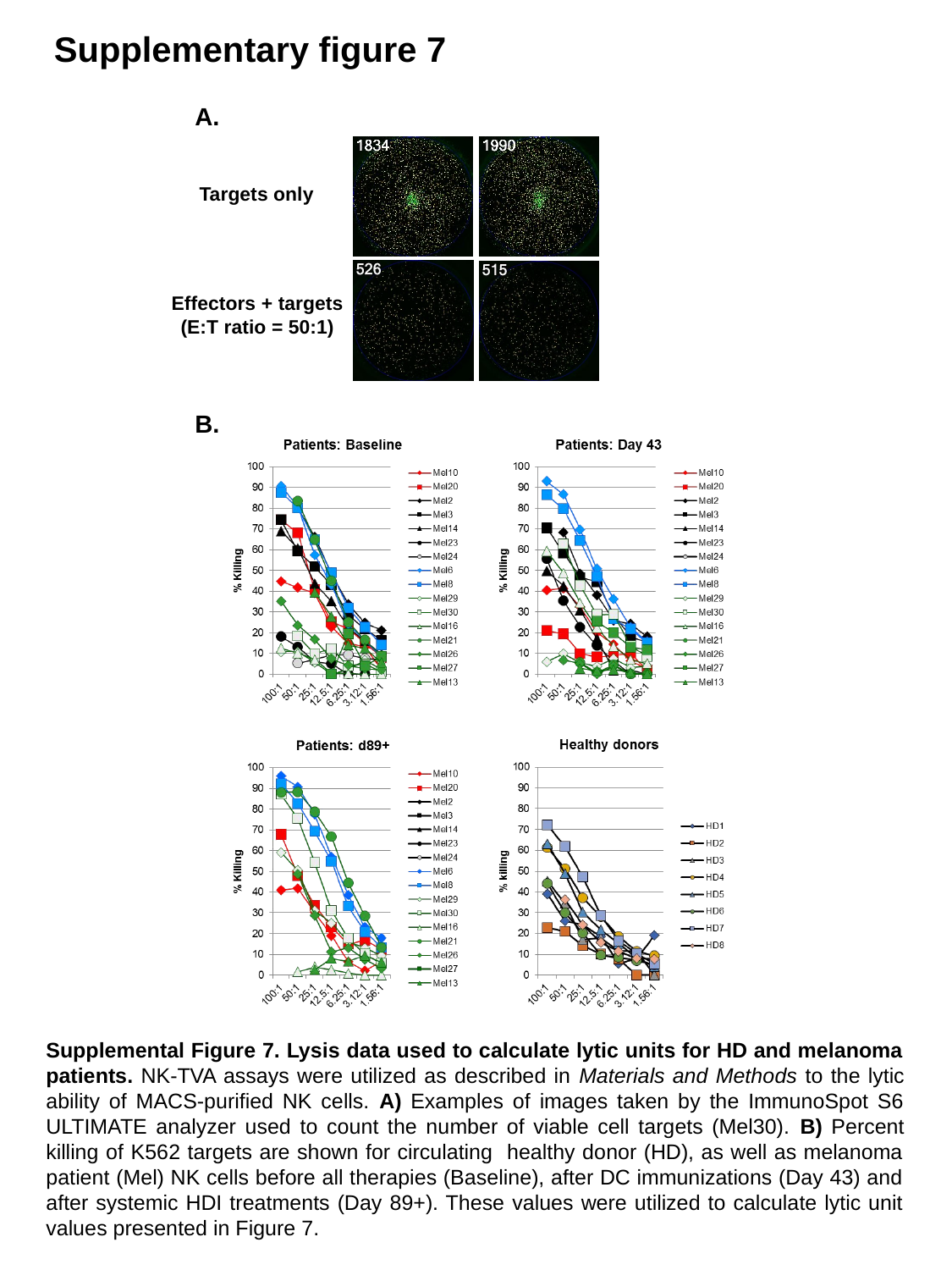

Supplementary figure 7
A.
Targets only
Effectors + targets
(E:T ratio = 50:1)
B.
Supplemental Figure 7. Lysis data used to calculate lytic units for HD and melanoma patients. NK-TVA assays were utilized as described in Materials and Methods to the lytic ability of MACS-purified NK cells. A) Examples of images taken by the ImmunoSpot S6 ULTIMATE analyzer used to count the number of viable cell targets (Mel30). B) Percent killing of K562 targets are shown for circulating healthy donor (HD), as well as melanoma patient (Mel) NK cells before all therapies (Baseline), after DC immunizations (Day 43) and after systemic HDI treatments (Day 89+). These values were utilized to calculate lytic unit values presented in Figure 7.

## Slide 10
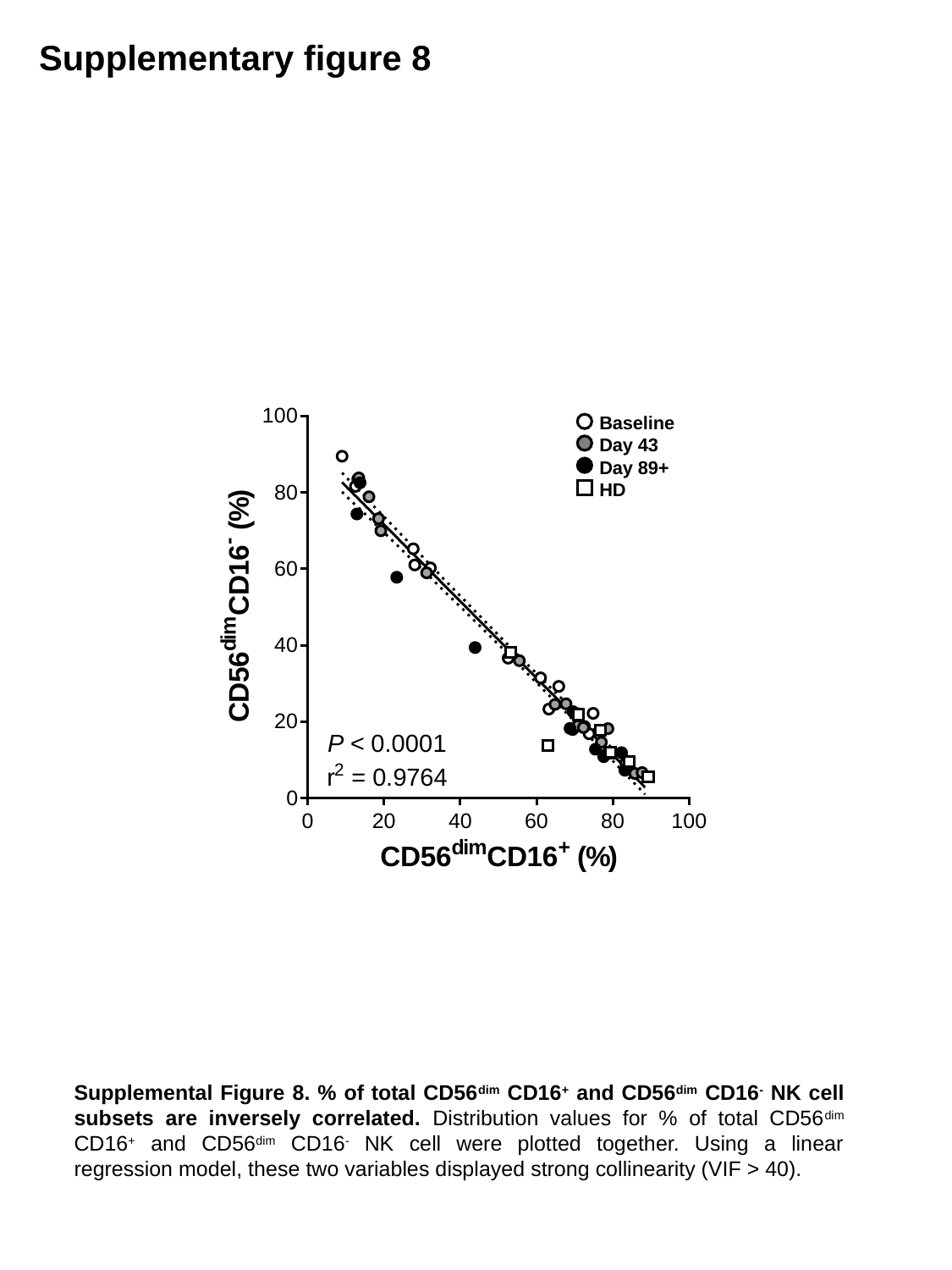

Supplementary figure 8
Baseline
Day 43
Day 89+
HD
Supplemental Figure 8. % of total CD56dim CD16+ and CD56dim CD16- NK cell subsets are inversely correlated. Distribution values for % of total CD56dim CD16+ and CD56dim CD16- NK cell were plotted together. Using a linear regression model, these two variables displayed strong collinearity (VIF > 40).

## Slide 11
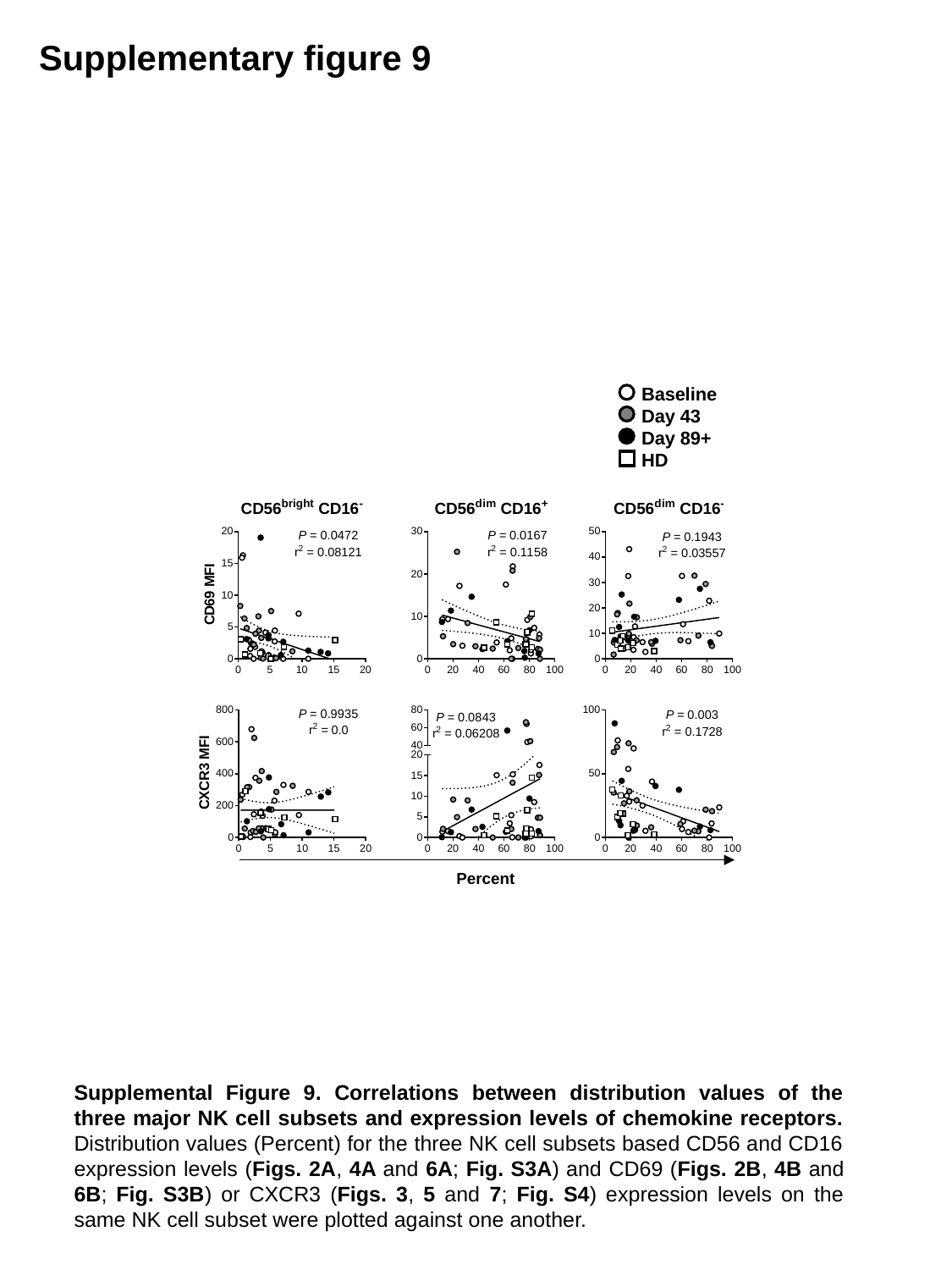

Supplementary figure 9
Baseline
Day 43
Day 89+
HD
Percent
Supplemental Figure 9. Correlations between distribution values of the three major NK cell subsets and expression levels of chemokine receptors. Distribution values (Percent) for the three NK cell subsets based CD56 and CD16 expression levels (Figs. 2A, 4A and 6A; Fig. S3A) and CD69 (Figs. 2B, 4B and 6B; Fig. S3B) or CXCR3 (Figs. 3, 5 and 7; Fig. S4) expression levels on the same NK cell subset were plotted against one another.

## Slide 12
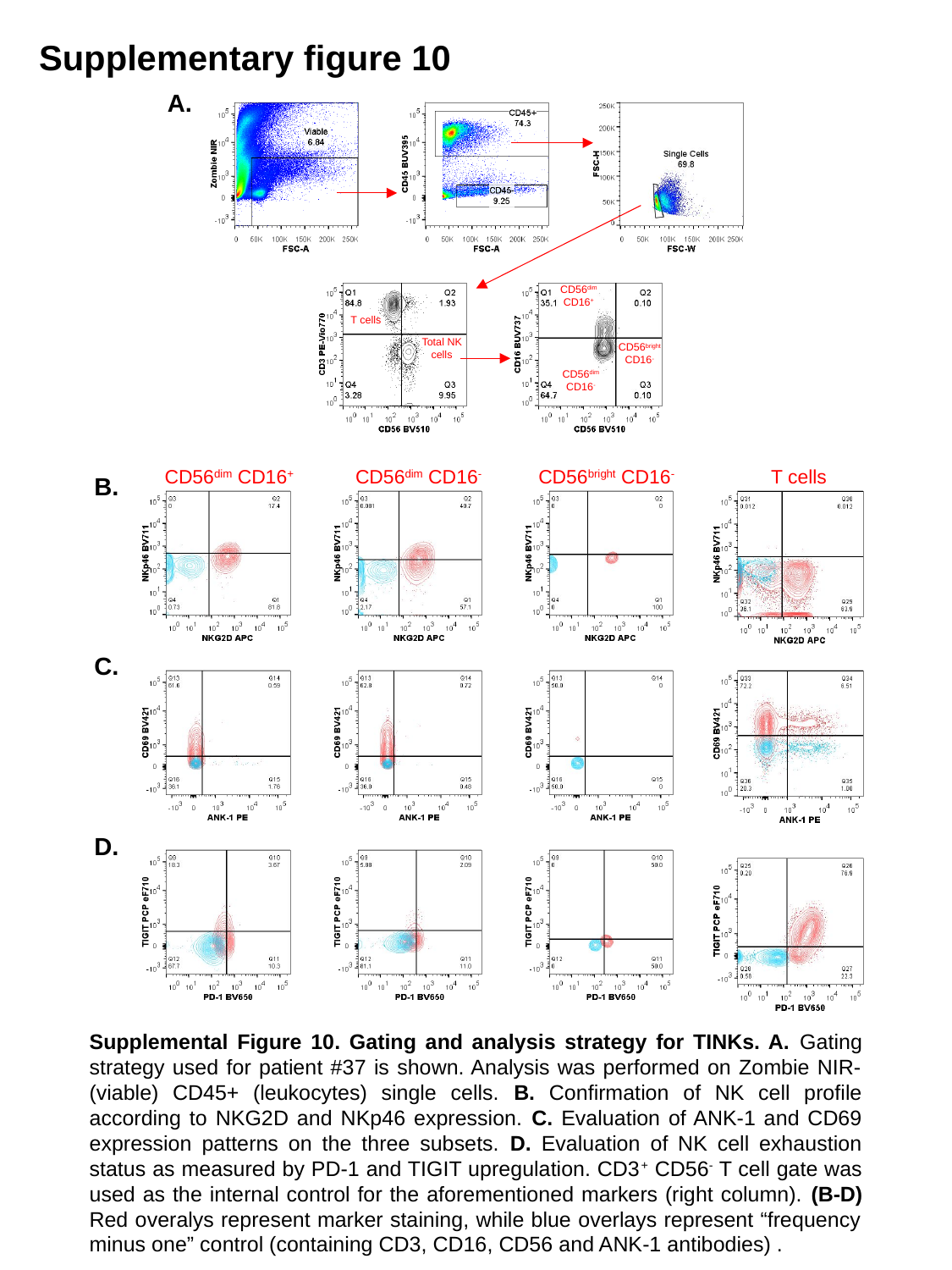

Supplementary figure 10
A.
T cells
Total NK cells
CD56dim CD16+
CD56bright CD16-
CD56dim CD16-
CD56dim CD16+
CD56dim CD16-
CD56bright CD16-
T cells
B.
C.
D.
Supplemental Figure 10. Gating and analysis strategy for TINKs. A. Gating strategy used for patient #37 is shown. Analysis was performed on Zombie NIR- (viable) CD45+ (leukocytes) single cells. B. Confirmation of NK cell profile according to NKG2D and NKp46 expression. C. Evaluation of ANK-1 and CD69 expression patterns on the three subsets. D. Evaluation of NK cell exhaustion status as measured by PD-1 and TIGIT upregulation. CD3+ CD56- T cell gate was used as the internal control for the aforementioned markers (right column). (B-D) Red overalys represent marker staining, while blue overlays represent “frequency minus one” control (containing CD3, CD16, CD56 and ANK-1 antibodies) .

## Slide 13
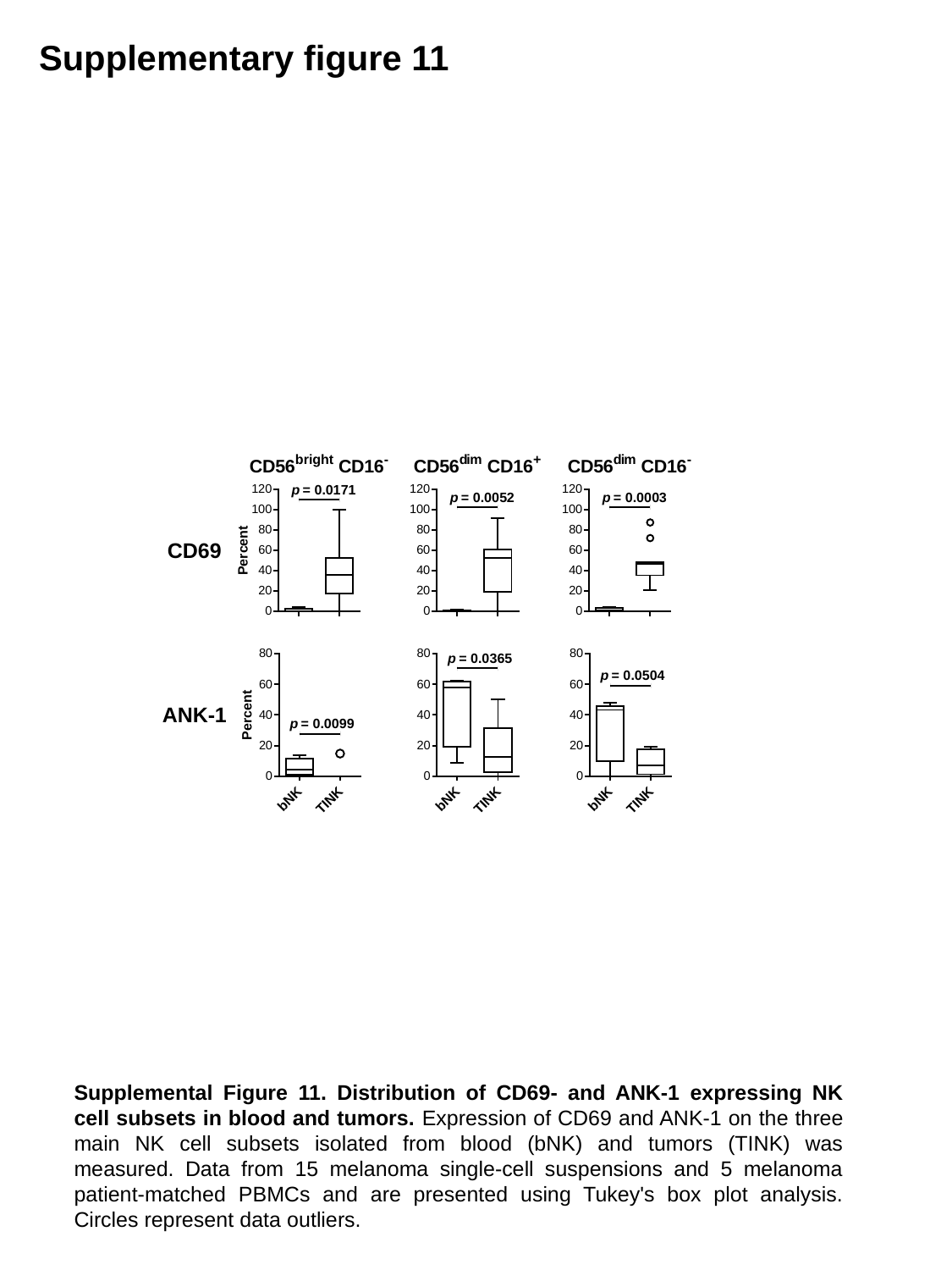

Supplementary figure 11
CD69
ANK-1
Supplemental Figure 11. Distribution of CD69- and ANK-1 expressing NK cell subsets in blood and tumors. Expression of CD69 and ANK-1 on the three main NK cell subsets isolated from blood (bNK) and tumors (TINK) was measured. Data from 15 melanoma single-cell suspensions and 5 melanoma patient-matched PBMCs and are presented using Tukey's box plot analysis. Circles represent data outliers.
